# Effective prediction of potential ferroptosis critical genes in clinical colorectal cancer

**DOI:** 10.3389/fonc.2022.1033044

**Published:** 2022-10-17

**Authors:** Hongliang Huang, Yuexiang Dai, Yingying Duan, Zhongwen Yuan, Yanxuan Li, Maomao Zhang, Wenting Zhu, Hang Yu, Wenfei Zhong, Senling Feng

**Affiliations:** ^1^ Department of Pharmacy, The Third Affiliated Hospital of Guangzhou Medical University, Guangzhou, China; ^2^ Guangdong Provincial Key Laboratory of Major Obstetric Diseases, Guangzhou, China; ^3^ School of Pharmaceutical Sciences, Guangzhou Medical University, Guangzhou, China; ^4^ School of Pharmacy, Guangdong Pharmaceutical University, Guangzhou, China; ^5^ The Fifth Affiliated Hospital of Guangzhou Medical University, Guangzhou, China

**Keywords:** bioinformatics, colon cancer, ferroptosis, differentially expressed genes, data mining, lipid peroxidation

## Abstract

**Background:**

Colon cancer is common worldwide, with high morbidity and poor prognosis. Ferroptosis is a novel form of cell death driven by the accumulation of iron-dependent lipid peroxides, which differs from other programmed cell death mechanisms. Programmed cell death is a cancer hallmark, and ferroptosis is known to participate in various cancers, including colon cancer. Novel ferroptosis markers and targeted colon cancer therapies are urgently needed. To this end, we performed a preliminary exploration of ferroptosis-related genes in colon cancer to enable new treatment strategies.

**Methods:**

Ferroptosis-related genes in colon cancer were obtained by data mining and screening for differentially expressed genes (DEGs) using bioinformatics analysis tools. We normalized the data across four independent datasets and a ferroptosis-specific database. Identified genes were validated by immunohistochemical analysis of pathological and healthy clinical samples.

**Results:**

We identified DEGs in colon cancer that are involved in ferroptosis. Among these, five core genes were found: *ELAVL1*, *GPX2*, *EPAS1*, *SLC7A5*, and *HMGB1*. Bioinformatics analyses revealed that the expression of all five genes, except for *EPAS1*, was higher in tumor tissues than in healthy tissues.

**Conclusions:**

The preliminary exploration of the five core genes revealed that they are differentially expressed in colon cancer, playing an essential role in ferroptosis. This study provides a foundation for subsequent research on ferroptosis in colon cancer.

## Introduction

Colon cancer is one of the most common and aggressive cancers worldwide, with high morbidity and poor prognosis. It is considered one of the most severe cancers, along with lung, prostate, and breast cancers (1). Up to 20% of colon cancers may be determined by genetic alterations and are primarily inherited diseases caused by overexpression of oncogenes and inactivation of tumor suppressor genes. Therefore, colon cancer is also influenced by several factors, including age and diet (2). This is because dietary factors can contribute to genetic changes or regulate other processes in cancer development. For instance, meat and fat seemingly increase the formation of free radicals in the intestine, primarily reactive oxygen species (ROS) (3). Free radicals, including oxygen radicals and lipid degradation products (i.e., 4-hydroxyalkenes and aldehydes), are genotoxic (4). Colon cancer is associated with reduced immune cytotoxicity (5) and diminished T-cell infiltration (6). Treatment for this cancer includes traditional surgery, radiotherapy, and chemotherapy, as well as risk factor modification, screening prevention, early diagnosis, and rapidly evolving immunotherapy (7). Adjuvant therapy may provide a survival advantage for some patients with colon cancer (e.g., stage III or II). However, not all patients benefit from these treatments (8). Therefore, it is necessary and urgent to explore new biomarkers and molecular mechanisms for targeted therapy of colon cancer to significantly improve patient prognosis and reverse cancer drug resistance.

Cell death is a fundamental physiological process for cell development, aging, and tissue homeostasis, which are hallmarks of cancer. Ferroptosis, a novel term created in 2012 by Dr. Brent R. Stockwell, describes a non-apoptotic form of programmed cell death dependent on intracellular iron (9). It is morphologically and biochemically distinct from other types of cell death and is characterized by an iron-dependent mechanism of intracellular lipid peroxidation induced by ROS. Ferroptosis is an essential cell death pathway that plays a crucial role in various diseases, including cancer, cardiovascular, and neurological diseases. In some cases, tumor metabolic reprogramming is associated with sensitivity to ferroptosis (10–12). Thus, a better understanding of the mechanisms underlying ferroptosis and the consequent development of new therapeutic strategies for treating tumor drug resistance would favor human health.

Bioinformatics and other computational tools, including artificial intelligence, play critical roles in medical care (13). We used a bioinformatics approach to preliminary explore essential genes with a role in ferroptosis in colon carcinogenesis. We applied data mining and data analysis techniques to screen for differentially expressed genes (DEGs) in pathological and healthy tissues of patients with colon cancer. These DEGs were then intersected with ferroptosis-related genes from the ferroptosis-specific database yielding critical genes associated with ferroptosis in colon cancer using various bioinformatics methods. In addition, we selected clinical tissue samples to validate these genes by immunohistochemistry. Our results contribute to the preliminary understanding and exploration of key genes that regulate ferroptosis in colon cancer, providing new ideas for targeted clinical therapy.

## Materials and methods

### Colon cancer-related gene expression and clinical data collection and processing

Gene expression profiles and related clinical data were obtained from a public database. Gene Expression Omnibus (GEO; https://www.ncbi.nlm.nih.gov/geo/), a gene expression database established by the National Center for Biotechnology Information (NCBI), contains gene expression data, including gene chips and high-throughput sequencing data. The microarray expression datasets, GSE41328, GSE44076, GSE110223, and GSE110225, were downloaded from the GEO database, and DEGs were accessed by comparing healthy colon tissue with colon cancer tissue (14). Dataset GSE41328 enclosed five colorectal adenocarcinomas and matched healthy colon tissues analyzed using the GPL570 array platform and Affymetrix HG-U133-Plus-2.0 microarrays. Dataset GSE44076 was acquired using Affymetrix Human Genome U219 Arrays on the GPL13667 platform to obtain gene expression profiles of paired healthy adjacent mucosal and tumor samples from 98 patients and 50 healthy individuals. GSE110223 and GSE110225 datasets contained DEGS of tumors and healthy colon tissue specimens, obtained by extracting total RNA using the GPL96 platform and further processing on the Affymetrix microarray platform HG-U133A.

All datasets were independent of each other and underwent the same processing. First, we download series matrix file(s) in the TXT format from each dataset to obtain the expression profile. To merge multiple data sets, we first merged the datasets using the R package inSilicoMerging, eliminating batch effects with the COMBAT method. Subsequently, we log_2_-transformed the data using the R package limma (version 3.40.6), a differential expression screening method based on generalized linear models, utilizing the lmFit function for linear regression. We further applied the eBays function for computing moderated t-statistics, moderated F-statistic, and log-odds of differential expression by empirical Bayes moderation of the standard errors. Finally, we obtained the DEGs between different pathological groups and control groups and plotted the differential gene volcanoes and sample clustering diagrams. Due to the excessive size of the feature matrix, we used principal component analysis (PCA) for dimensionality reduction of the data using the R software (version). The R package stats (version 3.6.0) was applied to obtain the Z-score, and the prcomp function was used to reduce the dimensionality, yielding a reduced matrix that simplifies the analysis of each dataset, removes redundant features, reduces the number of characteristic attributes, and highlights the characteristic attributes under research.

### Ferroptosis and colon cancer co-regulated genes

Ferroptosis is a form of cell death that is morphologically and biochemically distinguished from apoptosis, classical necrosis, autophagy, and other forms of cell death. However, it also causes a decrease in cell number (15). Ferroptosis-related genes were identified by searching the keyword *ferroptosis* in the FerrDb database [http://www.zhounan.org/ferrdb; (16)], the first database containing regulators and markers of disease-associated ferroptosis. To identify co-regulated genes between the colon cancer-related DEGs and ferroptosis-related genes, all processed datasets were analyzed using the Venny 2.1.0 online software (https://bioinfogp.cnb.csic.es/tools/venny/index.html). The enriched pathways for these common genes were analyzed using Metascape [http://metascape.org; (17)] and WebGestalt [http://www.webgestalt.org; (18)] and visualized using the ggplot2R package.

### Colon cancer and ferroptosis biological networks

Weighted correlation network analysis (WGCNA) is a systems biology method that describes genetic associations between samples and can be applied to identify highly synergistic sets of genes and biomarker genes or therapeutic targets. It is based on the endogeneity of the gene set and the association between the gene set and phenotype. Therefore, we performed WGCNA to identify common biological networks between colon cancer and ferroptosis. First, we calculated the median absolute deviation (MAD) of each co-regulated gene found using the methodology described in Section 2.2 through their expression profiles. Subsequently, we eliminated the top 50% of the smallest genes, removing the genes and samples with zero variation using the good Samples Genes function using the R package. Next, the WGCNA package was also used to construct a scale-free co-expression network. Pearson’s correlation matrixes and average linkage method were performed for all pairwise genes. A weighted adjacency matrix was constructed using the power function A_mn_ = |C_mn_|^β^, where A_mn_ is the adjacency between gene *m* and gene *n*; C_mn_ is the Pearson’s correlation between gene_m_ and gene_n_; *β* is a soft-thresholding parameter that can emphasize the strong correlations between genes. After a power of 18, the adjacency was transformed into a topological overlap matrix (TOM), a network gene pairing with the sum of the adjacencies of all other genes. Using the TOM, we measured the connectivity of the gene network and the corresponding dissimilarity (1-TOM).

The network type is unsigned, implying that genes within a module are highly correlated in an undirected network. To classify genes with similar expression into gene modules, average linkage hierarchical clustering was performed based on the TOM dissimilarity, with a minimum gene dendrogram size (genome) of 30. To further analyze the modules, we calculated the module eigengenes dissimilarity by setting the sensitivity to 3, selected a cut-off line for the module dendrogram, and merged a number of modules. It is possible to perform module-phenotype correlation analysis for each module after clustering genes into modules, draw a module-phenotype correlation heat map, divide samples into normal and tumor phenotypes, analyze the correlation phenotypes of modules of different colors corresponding to different phenotypes and identify the module with the highest correlation with the phenotype of interest.

### Protein-protein interaction analyses

The co-regulated DEGs found in Section 2.2 were also analyzed for their protein-protein interactions (PPIs) with the aid of the STRING online platform [https://cn.string-db.org; (19)]. The STRING database contains experimentally identified and predicted PPIs. The integrated networks were downloaded in the TSV format and imported into the Cytoscape 3.8.2 software. Network analysis and filtering-based top 10 degree values were implemented using the plug-in cytohubba and network analyzer tools. The Cytoscape application MCODE was used for clustering analysis of the gene networks to plot critical modules. Significantly enriched pathways for each module gene were analyzed through the online tools Metascape and WebGestalt, retaining ferroptosis-associated modules. The top 10 genes with degree values were merged with genes in critical modules, and duplicated genes were removed. The Cytoscape application MCODE was used for clustering analysis of the gene networks to plot critical modules.

### Functional enrichment analyses

Pathway enrichment analyses were performed for the genes screened in the 2.3 and 2.4 sections. We used the Kyoto encyclopedia of genes and genomes (KEGG) application programming interface (API, https://www.kegg.jp/kegg/rest/keggapi.html) for the R package org.Hs.eg.db (version 3.1.0) to acquire the latest KEGG pathway and gene ontology (GO) annotations as reference. Genes enrichment analyses were performed using the R package clusterProfiler (version 3.14.3), with a minimum gene set of 5 and a maximum of 5000, p-value < 0.05, and FDR < 0.1. The Benjamini-Hochberg correction was applied to the p-values.

### Correlation analysis of survival time

The TCGA database (https://www.cancer.gov) and GEPIA 2.0. was used to integrate 2, 5, 9, and 10 years of survival time, survival status, gene expression data of colon cancer, and clinical information (20). The gene expression data of intersecting genes screened in Section 2.2 were used. The TCGA portal (21) and LinkedOmics were used to fit the data and filter the potential ferroptosis-related genes in colon cancer, according to the p-value ranking obtained from survival analysis combined with prognostic significance.

### Identification of key genes in colon tumors and healthy tissues

Genes in colon cancer tumors and healthy tissues were analyzed and compared using R packages, the TCGA and GTEx databases, and the online tools LinkedOmics, TCGA portal, and Sangerbox (22). LinkedOmics is a publicly available portal that includes multi-omics data from all 32 TCGA cancer types and ten clinical proteomics tumor analysis consortium (CPTAC) cancer cohorts (23). Data from the GTEx and TCGA datasets were normalized using the scale function in the R software, removing samples with null expression values. Further gene expression analysis of the TCGA pan-cancer and GTEx normalized samples was performed using the Wilcoxon test within the TCGA portal. Furthermore, differences in the expression of critical genes in different tissue sites in colon cancer samples (N:245) were calculated using R software (version 4.1.2) and the LinkedOmics database. The Kruskal-Wallis test was used to evaluate the significance of the DEGs. Final data analyses were visualized using multi-group box plots. We also performed mutation correlation analysis for key genes. The mutation annotation format (MAF) is a text format TCGA applies to store mutation annotation information. The MAF files of the TCGA database were used to identify differences in mutation frequencies in each set of samples by the chi-square test. The maftool R package was used to draw the waterfall plots.

Subsequently, we downloaded the uniformly normalized pan-cancer dataset TCGA TARGET GTEx (PANCAN, N = 19131, G = 60499) from the UCSC database (https://xenabrowser.net/), for mutual validation. Samples with no expression values or less than three replicates were filtered out, and log_2_ (x + 0.001)-transformed. We used R software (version 4.1.2) to calculate expression differences between healthy and pathological colon samples and applied a non-parametric test to evaluate the statistical significance. Split violin plots were plotted with the ggplot2 function. The significance of gene differential expression levels in different tumor stages (COAD: Stage I = 66, Stage II = 63, Stage III = 61, Stage N = 206) were calculated using the unpaired Student’s t-test; non-parametric tests were used to assess the statistical significance of differences between multiple samples. Graphs were drawn on the bioinformatics platform (https://www.bioinformatics.com.cn).

### Immune cells infiltration correlated with key genes in colon cancer

TIMER assesses the infiltration of six immune cell types (B cells, CD4^+^ T cells, CD8^+^ T cells, neutrophils, macrophages, and dendritic cells) using the TIMER algorithm (24). Based on this platform, the expression profiles of colon cancer-related genes obtained by the method described in Section 2.5 were mapped to Gene Symbol. Samples with expression levels of 0 were filtered out using the Timer method of the R package IOBR (version 0.99.9) (25). Each expression value was log_2_ (x + 0.001)-transformed, and significant relationships between essential genes and immune cells were calculated. Spearman’s correlation coefficient between core genes was calculated using the corr test function of the R package psych (version 2.1.6). The bioinformatics platform was used for data visualization.

### Immunohistochemical analysis of critical genes in colon samples

Colon tissue samples were obtained from the Third Affiliated Hospital of Guangzhou Medical University (Guangzhou, China). All samples were collected after obtaining informed consent from patients. The experiments were approved by the ethics committee of the Third Affiliated Hospital of Guangzhou Medical University (number: 2022-076).

**Figure 1:**
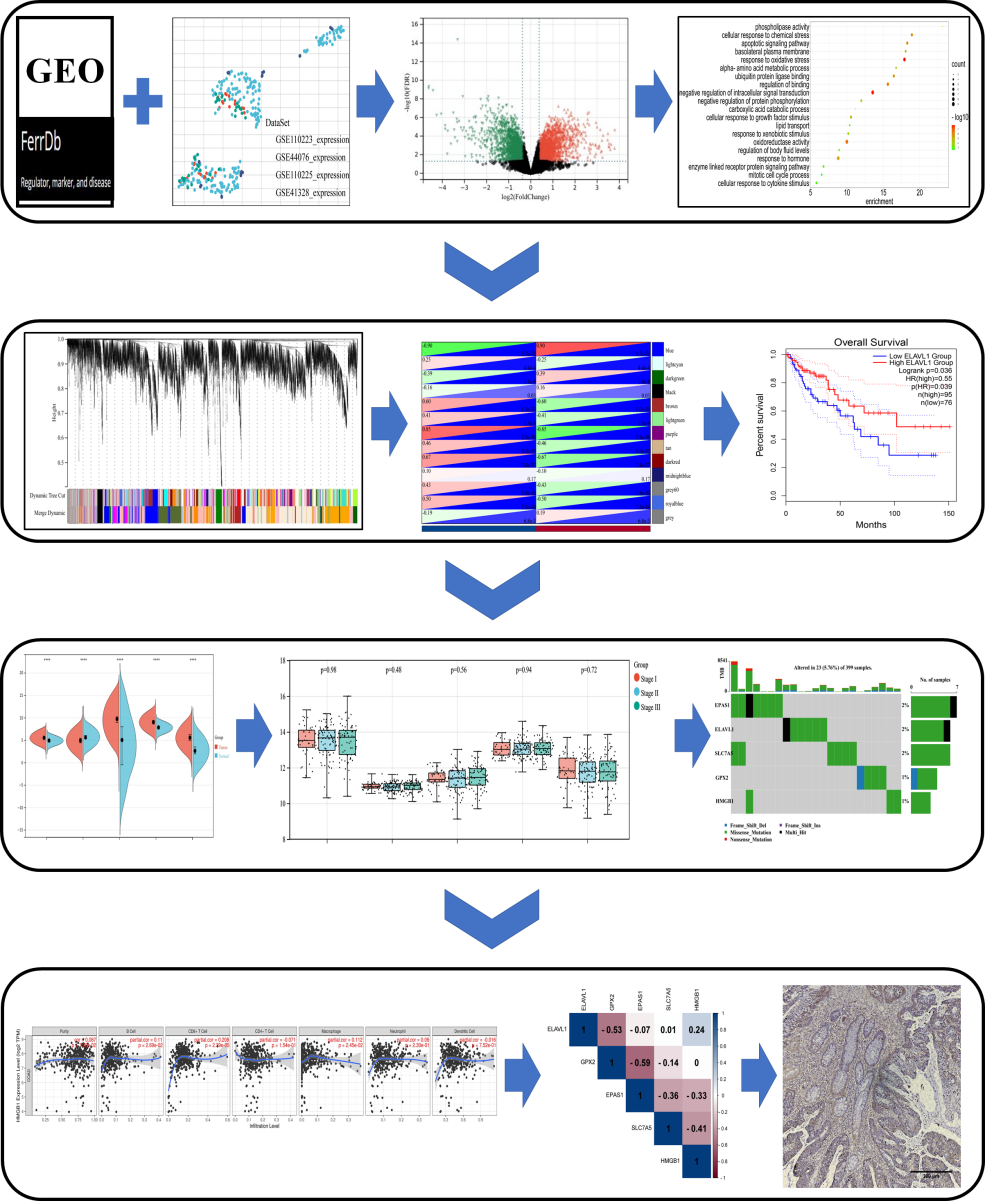
Schematic diagram of the study design. Differentially expressed genes (DEGs) associated with ferroptosis were identified in the GEO dataset and FerrDb database. Based on these DEGs, a study was conducted in the Metascape, WebGestalt database for enrichment analysis, and WGCNA and PPI analysis, and were analyzed by Cox survival analysis to obtain the key genes. The core genes were evaluated in multiple databases for gene characterization, downstream functional enrichment, mutational analysis and immune cell infiltration associated with ferroptosis. Validation of core gene expression by clinical samples from colon cancer patients.

All samples were oven baked at 70°C for 1 h. Next, xylene (I, II), absolute ethanol, 95% ethanol, and 75% ethanol were added in this order for dewaxing. Antigen repairing of tissue sections was performed to fully expose antigenic sites using 0.01 M citrate buffer in a 95°C water bath for 15 min, followed by natural cooling to room temperature. SP Rabbit & Mouse HRP kits (DAB) were purchased from CWBIO Co., Ltd. (Lot. 25121). Endogenous peroxidase blocking solution and standard sheep serum working solution were added and incubated at room temperature. Antibodies were diluted to the appropriate concentration and incubated overnight at 4°C. Biotin-labeled sheep anti-rabbit or mouse secondary antibodies, and HRP-labeled streptavidin were added dropwise and incubated at room temperature for 15 min. Next, DAB color development solution and hematoxylin were added dropwise. Films were sealed and stored after dehydration and air-drying and photographed under an inverted fluorescence microscope (Nikon ECLIPSE Ti2). The design diagram in this paper is shown in **Figure 1** for reference.

## Results

### Colon cancer alters genes expression profiles relative to healthy tissue

**Figure 2:**
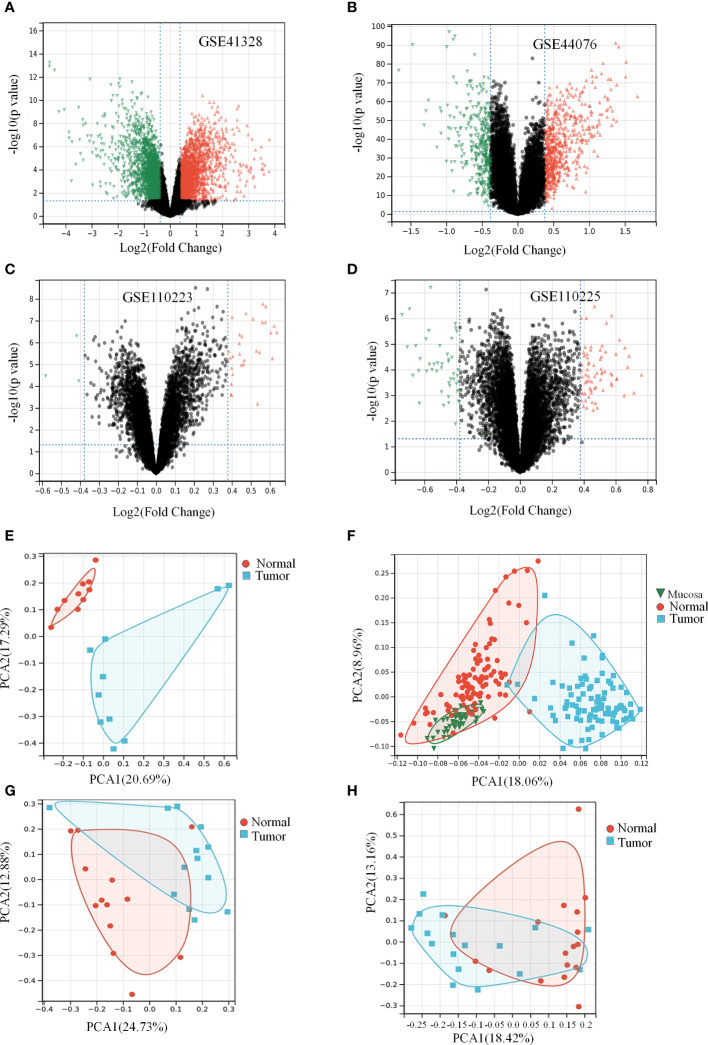
Colon cancer differentially causes the expression of genes in a certain extent. (**Table 1**) Expression differences of genes in data set**. (A, E)** Differential gene volcano plot and PCA analysis plot of GSE41328 dataset. **(B, F)** Differential gene volcano plot and PCA analysis plot of GSE44076 dataset. **(C, G)** Differential gene volcano plot and PCA analysis plot of GSE110223 dataset. **(D, H)** Differential gene volcano plot and PCA analysis plot of GSE110225 dataset.

As shown in the box plots and density plots, the sample distributions of each dataset differed significantly. In addition, the UMAP plots showed that samples of each dataset clustered together individually, suggesting a batch effect (**Supplementary Figure 1**). After eliminating the batch effect, given the size of the dataset, we performed the same analysis on each dataset individually and finally merged the results. DGEs between healthy and colon cancer samples with more than 1.3-fold change (logFC > 0.3) and p-value < 0.05 obtained from all databases and analysis are shown in **Figures 2A–D** and **Table 1** (26). The clustering results revealed two main clusters (**Supplementary Figure 2**). PCA facilitates the observation and mining of information from healthy vs. pathological tissue samples and visualizes the characteristics of both sets. As displayed in **Figures 2E–H**, the scatter points corresponding to the samples of the four groups overwhelmingly converged, indicating that the repetition between groups was relatively good and sample data were similar. Notably, the volcanoes plot in **Figure 2E** did not overlap, suggesting that the samples were well differentiated. Although samples illustrated in **Figures 2F–H** overlapped to some extent, the overlap was not significant, and the samples were not substantially similar. In summary, we hypothesize that colon cancer alters the gene expression profiles compared to healthy colon tissue.

**Table 1:** Number of differentially expressed genes and background in the four data sets.

	GSE41328		GSE44076		GSE110223		GSE110225
sample number	20		246		26		34
platform	GPL570		GPL13667		GPL96		GPL96&GPL570
molecule	total RNA		total RNA		total RNA		total RNA
source	Normal, matchednormal colon tissue		Healthy colon mucosa cells		normal adjacent sample		normal adjacent sample
	Tumor, colon adenocarcinoma		Normal distant colon mucosa cells		primary colorectal adenocarcinoma		primary colorectal adenocarcinoma
			Primary colon adenocarcinoma cells				
Submission date	3-0ct-12		5-Feb-13		6-Feb-18		6-Feb-18
Last update date	25-Mar-19		31-Aug-21		6-Mar-19		25-Mar-19
Contact name	Chieh-Chun Chen		Victor Moreno		Aristotelis Chatziioannou		Aristotelis Chatziioannou
ZIP/Postal code	61801		8908		11635		11635
Country	USA		Spain		Greece		Greece
Number (l.3-fold change, significance threshold <0.05)	Up- regulation	Down- regulation	Up-regulation	Down- regulation	Up- regulation	Down- regulation	Down- regulation
	2319	2442	639	364	31	3	46

### DEGs are engaged in biological processes and signaling pathways associated with ferroptosis

**Figure 3:**
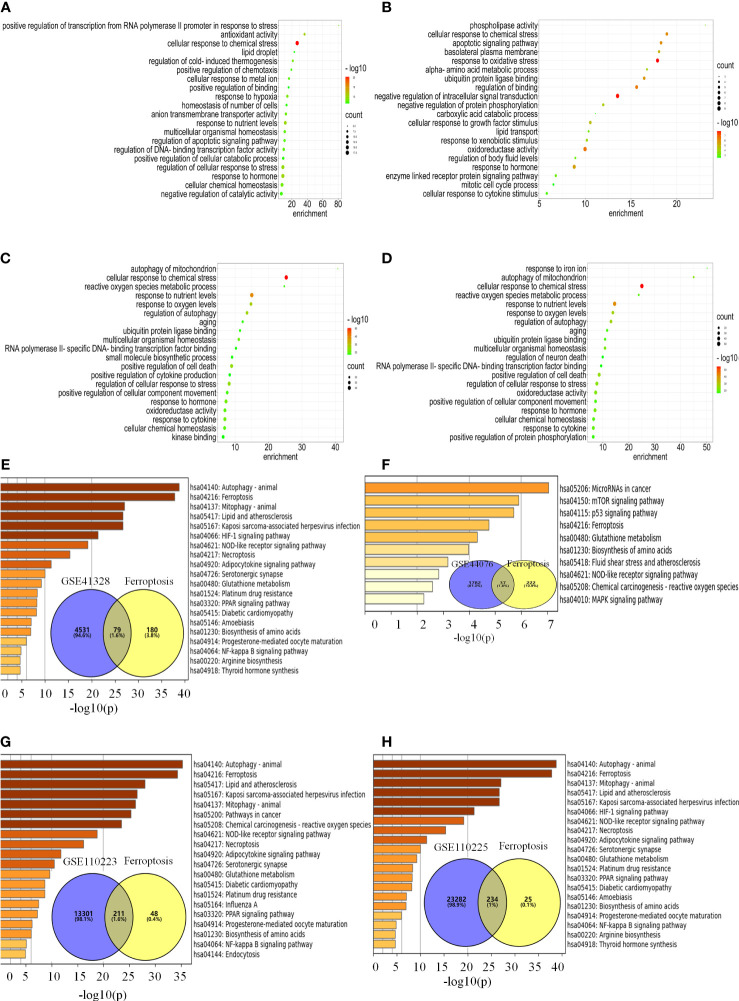
DEGs are engaged in biological processes and signaling pathways associated with ferroptosis. **(A–H)** Analysis of the pathway for ferroptosis-related differential genes for each data set. **(A–D)** Enrichment analysis of biological processes associated with ferroptosis-related differential genes for each data set. **(E–H)** Venn diagrams and enrichment analysis of the KEGG pathway for ferroptosis-related differential genes for each data set.

In the FerrDb database, 259 ferroptosis genes were retrieved, including drivers (108), suppressors (69), and markers (111), among which 28 were annotated with multiple functions. There were 79, 37, 211, and 234 co-regulated genes between ferroptosis and colon cancer datasets (**Figures 3E–H**). The enrichment analysis revealed that the ferroptosis-related DEGs were enriched in biological processes, such as cellular response to chemical stress, antioxidant activity, cellular response to metal ions, ROS metabolic process, response to oxidative stress, oxidoreductase activity, response to oxygen levels, positive regulation of cell death, and response to iron ions (**Figures 3A–D**). KEGG functional analysis revealed that signaling pathways, such as ferroptosis, p53, mTOR, PPAR, and HIF-1 signaling pathways, were significantly activated in colon cancer tissues (**Figures 3E–H**). Notably, the ferroptosis pathway is the main pathway involved, as confirmed by Metascape and WebGestalt. Therefore, DEGs are involved in biological processes and signaling pathways related to ferroptosis.

### Modular genes constructed by WGCNA might play an essential role in colon carcinogenesis

An essential process in the construction of a WGCNA is the selection of soft-thresholding power. GSE41328, GSE44076, GSE110223, and GSE110225 were identified to have soft thresholds of 18, 10, 9, and 18, respectively. The lowest power relative balanced scalar independence of the scale-free topological fit indices was 0.86, 0.85, 0.85, 0.85, and the average connectivities were 21.40, 56.60, 18.76, and 5.62 for the datasets GSE41328, GSE44076, GSE110223, and GSE110225, respectively (**Supplementary Figure 3**). This suggests high independence and co-expression similarity of gene expression profiles between each module and within the modules.

**Figure 4:**
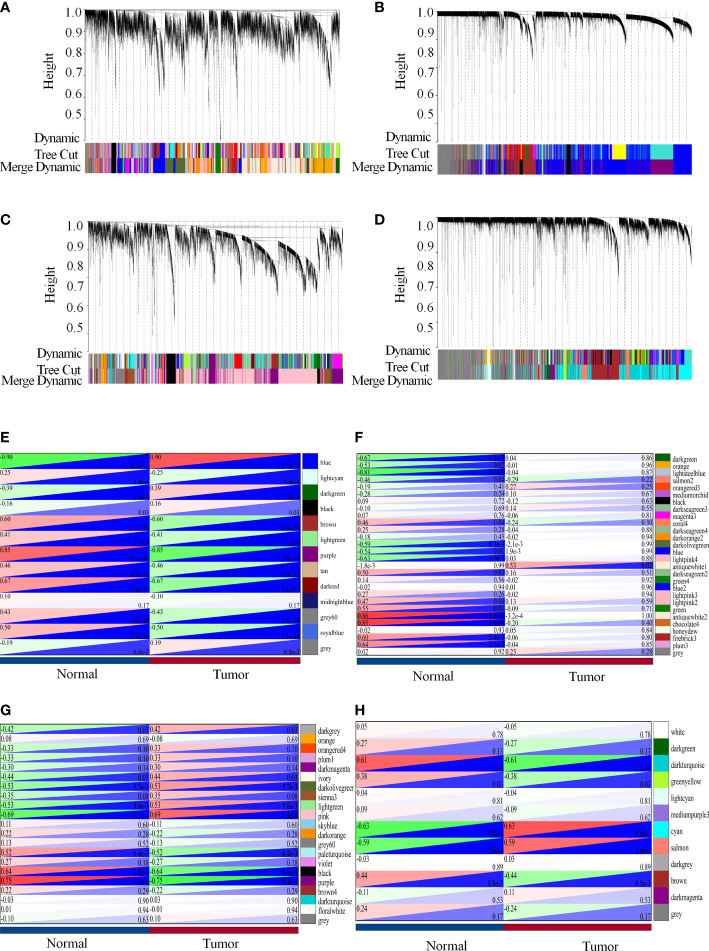
Modular genes constructed by WGCNA possibly play an essential role in colon carcinogenesis. **(A-H)** WGCNA analysis of each data set. **(A, E)** WGCNA analysis of the GSE41328 dataset. **(A)** Co-expression modules of the GSE41328 dataset; **(E)** Relationship between selected modules and clinical features. **(B, F)** WGCNA analysis of the GSE44076 dataset. **(B)** Co-expression modules of the GSE44076 dataset; **(F)** Relationship between selected modules and clinical features. **(C, G)** WGCNA analysis of the GSE110223 dataset. **(C)** Co-expression modules of the GSE110223 dataset; **(G)** Relationship of selected modules to clinical features. **(D, H)** WGCNA analysis of the GSE110225 dataset. **(D)** Co-expression modules of the GSE110225 dataset, and heat maps based on adjacency relationships. **(H)** Relationship of selected modules with clinical features.

Therefore, we merged modules with distances less than 0.25, resulting in modules for further WGCNA analysis, whereas gray modules were considered to be sets of genes that could not be assigned to any module (**Figures 4A–D**). GSE41328 obtained 28 modules, in which the antiquewhite2 module had 2275 genes, and the heat diagram was plotted according to the selected modules with clinical characteristics. The results showed that the modules strongly correlated with healthy colon tissue (**Figure 4E**). GSE44076 yielded 13 modules; the blue module had 3,404 genes, and the phenotype-associated heat map showed a high correlation between this module and colon cancer tissues (**Figure 4F**). Twenty-one modules were obtained for GSE110223, in which the pink module had 3,427 genes, and the module-phenotype correlation heat map showed a strong correlation between the module and colon cancer (**Figure 4G**). GSE110225 yielded 12 modules; the cyan module had 2634 genes, and a strong correlation was observed for the module-phenotype correlation heat map between this module and the occurrence of colon cancer (**Figure 4H**), indicating that the genes identified in the WGCNA analysis may play an important role in colon cancer.

### PPI network analysis revealed ferroptosis-related gene networks

Clustering analysis of the PPI networks of the GSE41328, GSE44076, GSE110223, and GSE110225 datasets yielded five, three, ten, and ten modules containing 59, 20, 632, and 643 genes, respectively (**Supplementary Figure 4**). The top 10 genes with degree values and module genes were merged, and duplicate values were removed, obtaining a total of 129 ferroptosis-related DEGs.

### Ferroptosis in colon cancer regulate a set of genes involved in the same pathway

**Figure 5:**
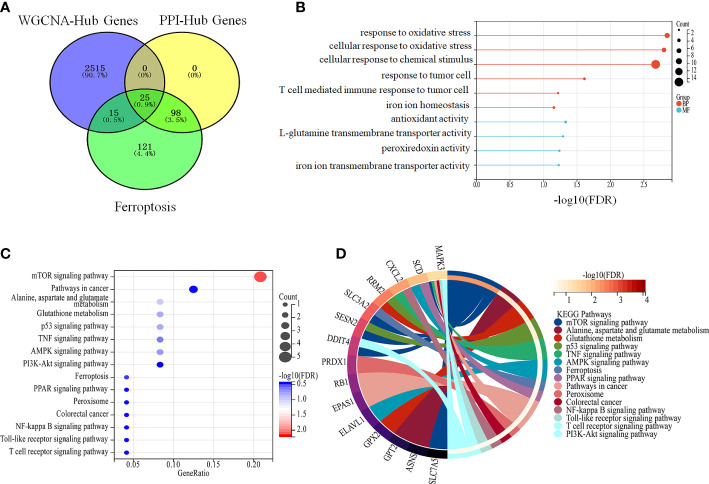
Intersecting genes are involved in ferroptosis in colon cancer. **(A)** Intersection of DEGs that were obtained from PPI, WGCNA analysis and ferroptosis genes **(B, C)** Enrichment analysis of intersecting genes. **(B)** Enrichment analysis of intersecting genes with biological processes related to ferroptosis. **(C)** Enrichment analysis of crossed genes with ferroptosis-related pathways. **(D)** Specific intercalating genes in ferroptosis-related pathways.

A total of 25 genes co-regulated during ferroptosis and colon cancer were identified by PPI and WGCNA analysis (**Figure 5A**). GO enrichment analysis showed that these 25 co-regulated genes were significantly enriched in cellular response to chemical stress, antioxidant activity, response to metal ions, ROS metabolic process, and other biological processes associated with ferroptosis (**Figure 5B**). Furthermore, the 25 genes were significantly enriched for ferroptosis pathway, p53 signaling pathway, and glutathione metabolism, which included genes such as those encoding glutathione peroxidase 2 (*GPX2*), endothelial PAS domain protein 1 (*EPAS1*), solute carrier family 7 member 5 (*SLC7A5*), and mitogen-activated protein kinase 3 (*MAPK3*; **Figures 5C, D**). These genes are potential marker candidates for ferroptosis in colon cancer.

### Ferroptosis genes inhibit the development and progression of colon cancer

**Figure 6:**
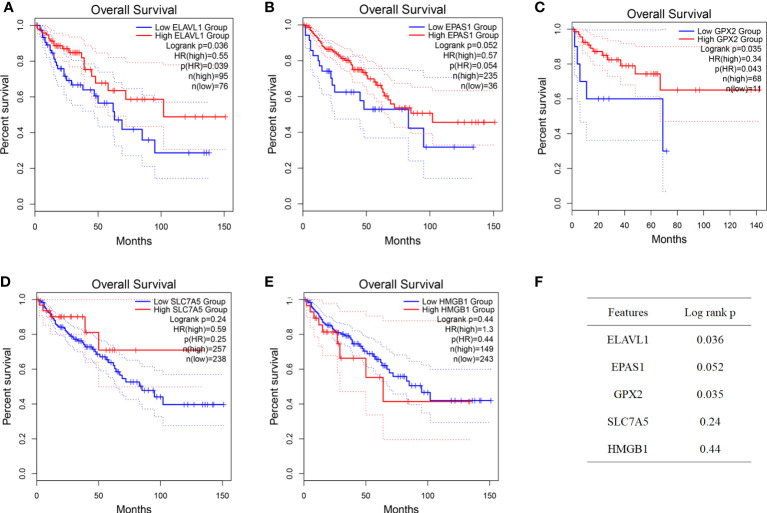
Identification of key genes. **(A–F)** Prognostic significance of key genes and their *P*-value.

**Figure 7:**
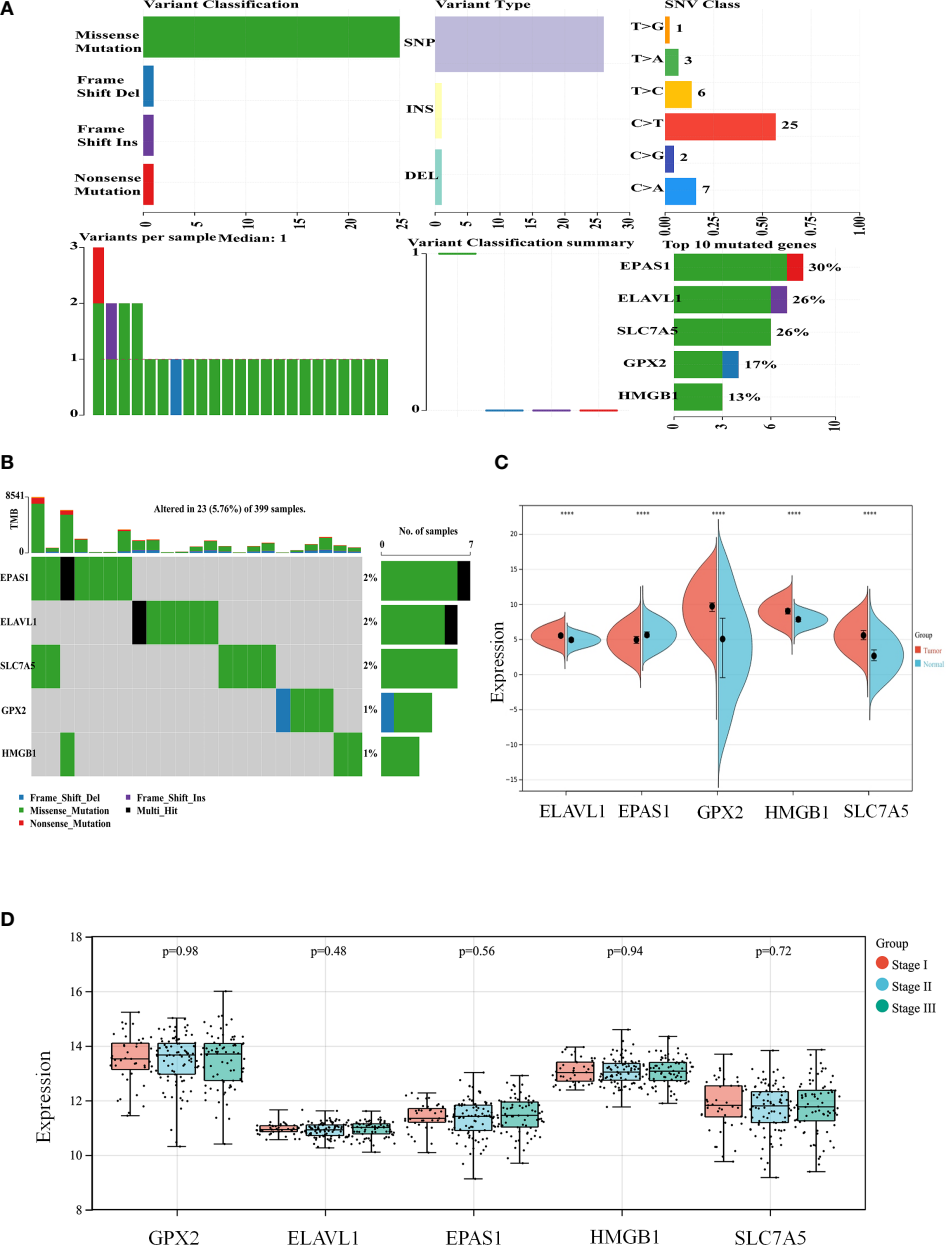
The expression of key genes plays an important role in the development and progression of colon cancer. **(A, B)** Mutation correlation analysis of key genes. **(C)** Expression of the key genes in colon cancer tissues and in normal colon tissues from the UCSC database of colon cancer expression profiles. **(D)** Expression of key genes in different tumor stages. ****P < 0.0001.

According to the colon cancer Cox regression test (**Table 2**) and Kaplan–Meier (K-M) curves of key genetic risk factors (**Figure 6**), the five most significant genes selected were: ELAV-like RNA-binding protein 1 (*ELAVL1*), *GPX2*, *EPAS1*, *SLC7A5*, and high mobility group box 1 (*HMGB1*). A total of 399 samples with detected mutations were analyzed to correlate the mutations with these core genes, of which 23 (5.76%) were included in the mapping samples. We identified mainly missense mutations, single nucleotide polymorphism (SNP), and single nucleotide variation (SNV) of C>T, in which *EPAS1* had the highest mutation frequency (**Figures 7A, B**).

**Table 2:** Name and P-value ranking of the 25 genes that intersect with PPI, WGCNA and ferroptosis genes.

Gene name	p-value	Gene name	p-value
ELAVL l	9.93E-O1	ASNS	4.47E-O1
GPT2	9.69E-O1	GDF15	4.16E-O1
MAPK3	9.27E-O1	AURKA	3.70E-O1
DDIT4	8.57E-01	SLC3A2	3. 57E-O1
GPX2	8.33E-O1	SLC1A4	3.46E-O1
EPAS1	8.27E-O1	RRM2	3.01E-01
TRIB3	7.74E-O1	STMN1	2.07E-O1
TFR2	7.33E-01	TXNIP	1.95E-01
SLC7A5	6.72E-O1	SCD	1.47E-01
PSAT1	6.57E-O1	WIPI1	1.02E-O1
HMGB1	6.03E-O1	CXCL2	5.85E-02
RB1	5 .89E-01	SESN2	5.18E-02
PRDX1	4.64E-01		

The split violin plot diagram revealed that the expression profiles of these core genes in different databases of colon cancer were higher in tumors than in healthy tissues, except for *EPAS1*, and the results were statistically significant (**Figure 7C**). The gene expression profiles in colon cancer were identified using TCGA and GTEx databases. Most tumor samples were distributed in stages I–III. However, bioinformatics analysis showed no statistically significant results in tumor stages (**Figure 7D**).

### Identification of ferroptosis-related genes by immune signature

**Figure 8:**
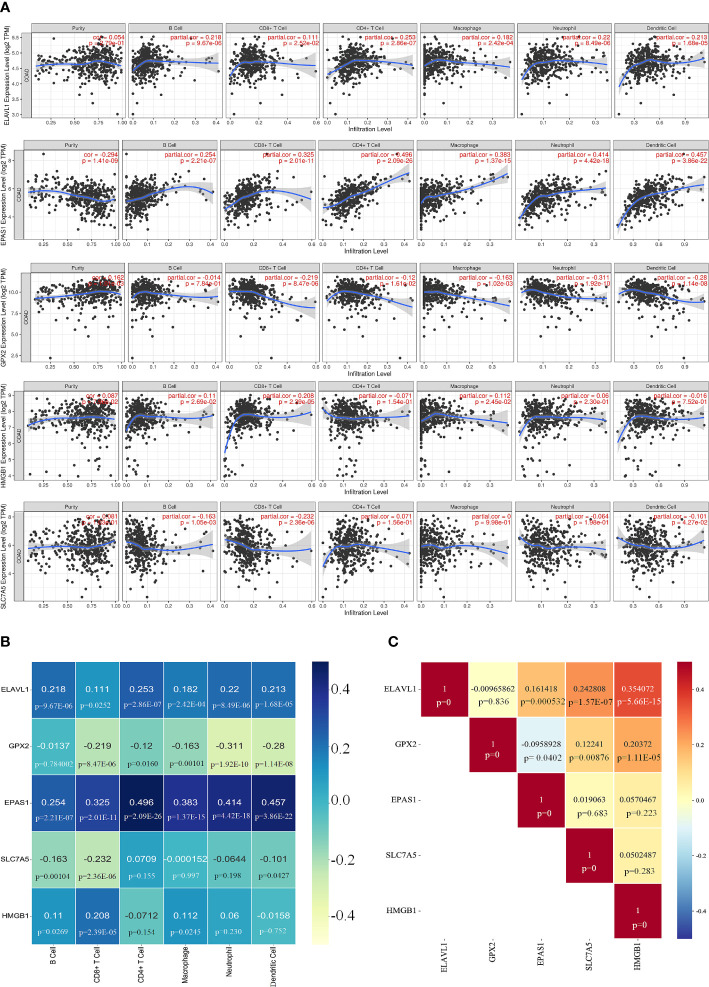
Correlation of key genes and immune cells. **(A, B)** Correlation of five key genes with six immune cell. **(C)** Significant relationship between each core gene.

The correlation between the five core genes and six immune cells was assessed using the TIMER platform. Expression of ELAVL1 and EPAS1 was positively correlated with all six immune cells analyzed, although a higher correlation was observed for CD4^+^ T cells. Interestingly, all six immune cells negatively correlated with GPX2, which strongly correlated with neutrophils. HMGB1 positively correlated with four immune cells except CD8^+^ T. SLC7A5 negatively correlated with CD8^+^ T cells (**Figures 8A, B**).

The significant relationship of the core genes was analyzed, showing that GPX2 was negative correlation with EPAS1; ELAVL1 did not correlate with GPX2; HMGB1 and ELAVL1 were positively correlated, and SLC7A5 was highly correlated with ELAVL1 (**Figure 8C** and **Supplementary Figure 6**). IFN-γ secreted by CD8^+^ T cells can inhibit systemic *XC* expression, thus promoting iron-induced death in tumor cells (27). The infiltration of colorectal cancer (CRC) by CD8^+^ T-expressing positive lymphocytes has been associated with a good prognosis (28).

### Immunohistochemical analysis

**Figure 9:**
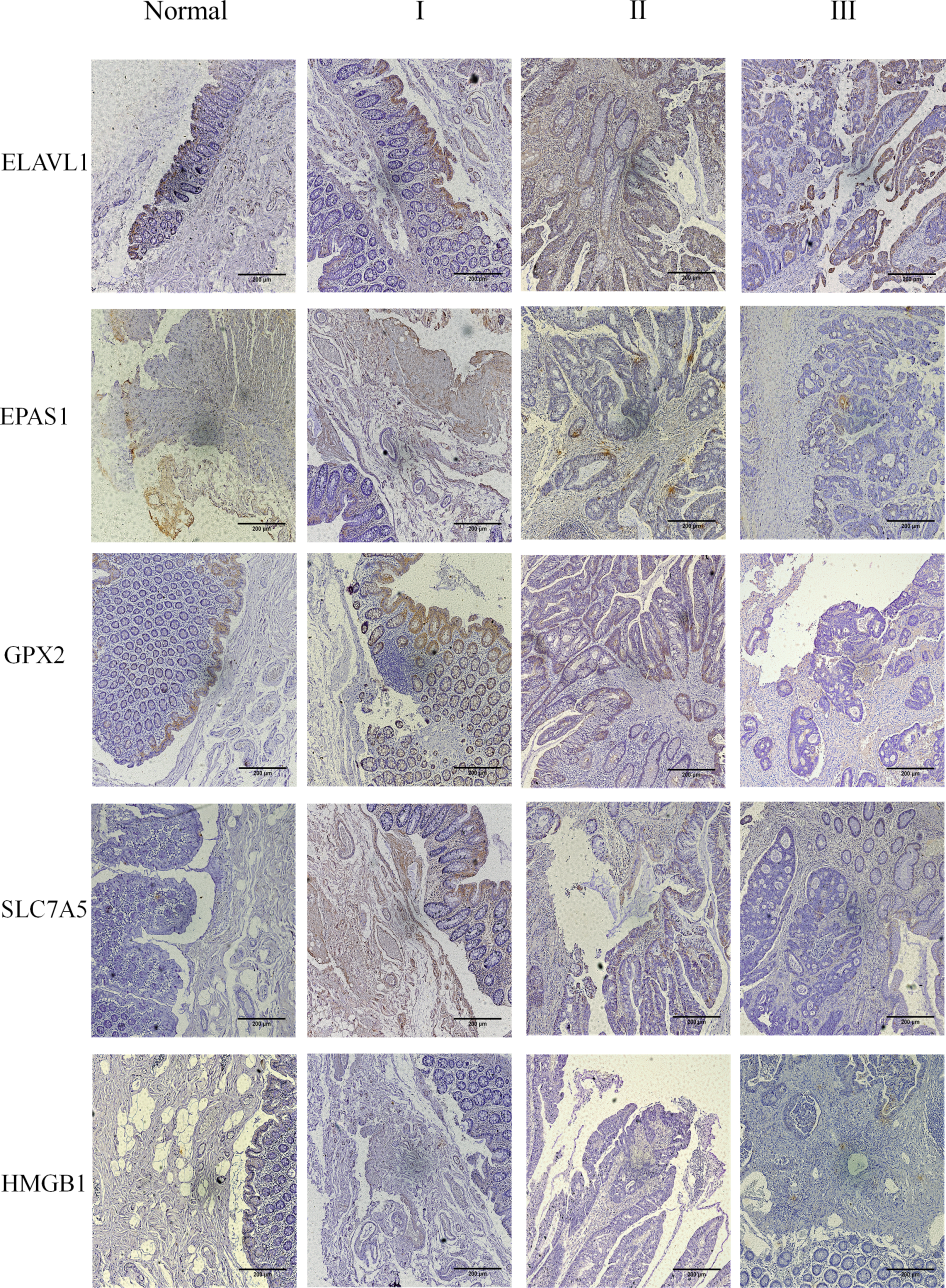
The five core genes were expressed in the tumor tissues of stage I–III. Clinical tissue samples were grouped in normal and tumor tissues (stage I–III), and the expression of the five key genes in the clinical samples was observed at multiples of 10X.

The results showed that the five core genes were expressed in stage I–III tumor tissues, as shown in **Figure 9** and **Supplementary Figure 5**. According to the color and quantity of positivity: ELAVL1 was expressed in stages I–III and was differentially expressed between tumor and normal tissues. The experimental results matched the results in **Figure 7C**. EPAS1 was downregulated in tumor tissues relative to normal tissues, with significant differences between stages I and III, and II and III. The experimental results were consistent with the results shown in **Figure 7C**. GPX2 was significantly upregulated in tumor tissues compared to normal tissues; there was no significant difference in stages I–III; experimental results validate the results shown in **Figures 7C, D**. SLC7A5 was significantly upregulated in tumor tissues relative to normal tissues; experimental results validated the results shown in **Figure 7C**. HMGB1 expression did not significantly differ between tumor and normal tissues. However, significant differences were observed between stages I and II and stages I and III. The experimental results were consistent with the results in **Figure 7C**.

## Discussion

Colon cancer is the third most common cancer worldwide and the leading cause of cancer-related death (29). Ferroptosis is a newly discovered form of regulated cell death governed by iron-dependent oxidative stress and lipid peroxidation (9, 30). Iron, one of the most abundant transition metals in the body, plays a pivotal role in several biochemical reactions (31). Nevertheless, excessive iron is detrimental to cells, and ROS generated by lipid peroxidation, iron accumulation, and mediated by the Fenton reaction play a critical role in ferroptosis (32). Iron exists primarily in two oxidation states in cells, ferrous (Fe^2+^) and ferric (Fe^3+^), which can accept and provide electrons. When overloaded with iron, catalytic Fe^2+^ is produced, causing a Fenton reaction that can convert hydrogen peroxide into toxic hydroxyl radicals (^-^OH). These free radicals attack lipids in the vicinity of membrane phospholipids. They can lead to polyunsaturated fatty acid (PUFAs) peroxidation and subsequent excessive ROS production, leading to DNA and protein damage and cellular ferroptosis (33, 34). Accordingly, ferroptosis is a novel, regulated form of cellular necrosis caused by excessive accumulation of ferrous iron and ROS (35).

In this study, we analyzed four datasets from public databases to identify DEGs between healthy and colon cancer tissues. We performed co-regulation, WGCNA, and PPI analyses with ferroptosis-related genes from the FerrDb database using bioinformatics. We identified five core genes, *ELAVL1*, *GPX2*, *EPAS1*, *SLC7A5*, and *HMGB1*, involved in the ferroptosis of colon cancer. The expression of these genes was shown to be altered in the colon during carcinogenesis and correlated with ferroptosis-related biological processes and signaling pathways. Moreover, according to *in silico* analysis and tissues immunohistochemical analysis, all genes except for EPAS1 were overexpressed in tumor tissues relative to healthy tissues.

The messenger ELAVL1 is a cancer-associated RNA-binding protein, also known as human antigen R. It is a ubiquitous member of the RBP family and acts as a positive regulator of RNA stability by stabilizing the 3´-untranslated region of the AU-rich cis-acting element (Ares) of the mRNA (36, 37). ELAVL1 targets include proto-oncogenes, growth factors, and invasive factors; thus, it plays an essential role in cancer (38). *ELAVL1* expression is higher in almost all cancer tissues than in healthy ones (39). Immunohistochemical analysis of paired tumor and healthy tissues revealed that ELAVL1 expression and cytoplasmic abundance positively correlated with tumor malignancy and advanced tumor stage, especially in colon cancer (40, 41). ELAVL1 plays a vital role in activating iron-phagocytic proteins and promoting ferroptosis (42), altering cellular responses to oxidative stress, proliferation, differentiation, and immune stimulation (43, 44). Exposure to ferroptosis inducers results in a significant increase in ELAVL1 protein expression by inhibiting the ubiquitin-proteasome pathway. Besides, *ELAVL1* overexpression appears to augment the production of autophagic vesicles, a potential mechanism for ELAVL1-promoted ferroptosis. However, *ELAVL1* downregulation through RNA interference (RNAi) results in ferroptosis resistance (45).

Hypoxia is a tumor microenvironment hallmark that contributes to tumorigenesis and drug resistance development, which help tumors evade cell death through multiple mechanisms (46). Hypoxia-inducible factor 2-alpha (HIF2α), encoded by the *EPAS1* gene, is an oxygen-sensitive component of HIF1 (47). EPAS1 plays a critical regulatory role in ROS production and lipid metabolism in various cells (48, 49). *EPAS1* expression leads to increased tumor growth and metastasis (50) and is significantly associated with the occurrence, development, and prognosis of colorectal cancer. In colorectal cancer, EPAS1 expression was negatively correlated with tumor grades, and mRNA expression of *EPAS1* was significantly lower in primary colorectal cancer tissues than in healthy tissues and correlated with advanced pathological stages III and IV and poor patient survival (51, 52). Furthermore, HIF-2α activation in colon cancer enhances oxidative cell death by increasing cellular iron levels (53). HIF-2α is selectively enriched in an acyl-CoA synthetase long-chain family member 4 (ACSL4)-dependent manner by polyunsaturated lipids that are required for lipid peroxidation and subsequent ferroptosis (54), which is promoted by lipid oxidation, ROS accumulation, and ferritin deposition (55).

The isozymes glutathione peroxidase 1 (GPX1) and GPX2 are the main enzymes responsible for hydrogen peroxide reduction in intestinal epithelial cells (56). GPX2 is loosely bound to the cytosolic side of the outer mitochondrial membrane and the matrix side of the inner mitochondrial membrane (57). In yeast, GPX2 counteracts the production of harmful ROS in the presence of PUFA, such as ROS produced in the mitochondria (58). The primary transcription factor that induces *GPX2* is the nuclear factor erythroid 2–related factor 2 (NRF2) (59). The expression profile of *GPX2* observed here is also likely transcriptionally regulated by NRF2. GPX2 knockdown is followed by NRF2-induced upregulation of GPX2 and cytochrome c oxidase 2 (COX2), revealing the suppression of inflammation-mediated cancer (60). In human colon cancer, lipid peroxidation is directly proportional to the cancer stage (61). Specific ROS, such as hydrogen peroxide, are secondary messengers in the tumor signaling pathway. GPX2 keeps intracellular hydrogen peroxide levels low, thus maintaining clone formation and tumor cell numbers (62). GPX2 was the first protein to be identified as a target of the Wnt pathway (63). In inflammation-triggered carcinogenesis, GPX2 and the Wnt pathway co-localize at the base of intestinal crypts and are induced by Wnt signaling. When the Wnt pathway is dysregulated, GPX2 may promote tumor growth (64, 65).

The DNA-binding protein HMGB1 is a ubiquitous transcriptional regulator in almost all eukaryotic cells and is highly conserved between species without sequence specificity (66, 67). The HMGB1 protein is localized in the nucleus and translocated to the cytoplasm or extracellular space when stimulated by cytokines, lipopolysaccharides, or hypoxia (68, 69). Significantly elevated HMGB1 protein levels in colon cancer tissues are associated with poor prognosis and the absence of extensive macrophage infiltration (70). Similarly, HMGB1 is released upon exposure to various ferroptosis inducers (71). The secreted HMGB1 induces the matrix metalloproteinase pathway and increases the invasiveness of cancer cells (72). Oxidative stress can be prevented by inhibiting ferroptosis through the NRF2/HO-1/HMGB1 axis (73). HMGB1 also regulates ferroptosis *via* the Ras-JNK/p38 pathway in leukemia (74).

The human SLC7A5 transporter protein, also known as LAT1, is a sodium-independent reverse transporter positively regulated by physical interaction with membrane cholesterol and intracellular ATP, providing essential amino acids to the cell and maintaining dynamic cellular homeostasis (75, 76). SLC7A5 is overexpressed in the plasma membrane of aggressive cancer cells, and its expression level correlates with poor patient prognosis (77). In a pathological correlation study of non-small cell lung cancer (NSCLC) clinical stages I−III, the 5-year survival rate of SLC7A5-positive patients was significantly lower than that of SLC7A5-negative patients (78). In addition, SLC7A5 is upregulated in cancer cells and exhibits high cancer specificity (79).

Our bioinformatics analysis showed no statistically significant results according to tumor stages. However, the expression of key genes was significantly different in tumor and normal tissues, as evidenced by statistical analysis of immunohistochemistry results, although the expression of some genes did not significantly differ between tumor stages. Overall, the immunohistochemical experiments verified the bioinformatic predictions of core genes in normal and pathological tissues. Nonetheless, the results across tumor stages differed from the bioinformatic results. Possible reasons for the discrepancy might be the selection of data sets, differences in algorithms, datasets, databases, and clinical sample heterogeneity.

The currently available treatment strategies against colon cancer, a leading cause of cancer-related deaths worldwide, provide a survival advantage for some patients. However, not all patients benefit from these treatments. Therefore, novel biomarkers and molecular mechanisms must be identified to develop targeted colon cancer therapy, significantly improving the disease prognosis and reversing cancer drug resistance. Ferroptosis, a newly discovered form of iron-dependent cell death, could help overcome tumor drug resistance and prevent cancer cell metastasis. To improve our understanding of the relationship between colon cancer and ferroptosis, we analyzed four datasets from public databases to identify DEGs between healthy and colon cancer tissues. We also performed co-regulation, WGCNA, and PPI analyses with ferroptosis-related genes from the FerrDb database. Our results suggest that five core genes, ELAVL1, GPX2, EPAS1, SLC7A5, and HMGB1, are involved in the ferroptosis of colon cancer cells. Finally, we validated the correlation of these genes with colon cancer using immunohistochemical analysis of pathological and healthy clinical samples, confirming the different expression of all five genes in tumor tissues and healthy tissues were confirmed. We believe our study contributes significantly to the literature because our results open new possibilities in cancer therapy, providing new candidates for further studies on colon-targeted therapy and cancer drug resistance.

As a novel possibility in cancer therapy, ferroptosis inducers can help overcome tumor drug resistance, preventing metastasis and the spread of cancer cells. Based on this, we initially explored key genes for ferroptosis in colon cancer and propose that the upregulation or downregulation of the five core genes identified here may be candidate targets for inducing ferroptosis in colon cancer cells. To further test this hypothesis, subsequent research on the ferroptosis pathways that involve these five genes in colon cancer is warranted. We believe this study provides a foundation for further research and new ideas for targeted therapy for colon tumors and cancer drug resistance.

## Data availability statement

The original contributions presented in the study are included in the article/**Supplementary Material**. Further inquiries can be directed to the corresponding authors.

## Ethics statement

The studies involving human participants were reviewed and approved by Ethics Committee of the Third Affiliated Hospital of Guangzhou Medical University(number: 2022-076). The patients/participants provided their written informed consent to participate in this study.

## Author contributions

SF and WZ designed the research. HH, YDa and YDu carried out the experiments and performed data analysis. ZY, WZ, WZhu, YL, MZ and HY participated part of the experiments. HH, YDa and YDu wrote the manuscript. SF and WZ revised the manuscript. All authors contributed to the article and approved the submitted version.

## Funding

This work was supported by National Natural Science Foundation of China (No.82104446); Guangdong Basic and Applied Basic Research Foundation (No.2019A1515111109); Guangdong Province Characteristic Innovation Project of universities (No.2022KTSCX100); Yiwen Talent Project; Academician He Lin New Medical Research Foundation (No.2021HLKY02).

## Acknowledgments

We would like to thank Editage (www.editage.cn) for English language editing.

## Conflict of interest

The authors declare that the research was conducted in the absence of any commercial or financial relationships that could be construed as a potential conflict of interest.

## Publisher’s note

All claims expressed in this article are solely those of the authors and do not necessarily represent those of their affiliated organizations, or those of the publisher, the editors and the reviewers. Any product that may be evaluated in this article, or claim that may be made by its manufacturer, is not guaranteed or endorsed by the publisher.

## Supplementary material

Supplementary Figure 1
**(A-D)** Merging of multiple data sets.

Supplementary Figure 2
**(A-D)** grouping cluster analysis plot of dataset**. (E-H)** sample cluster analysis plot of dataset.

Supplementary Figure 3
**(A-H)** Scalar independence and average connectivity of WGCNA analysis for each dataset.

Supplementary Figure 4
**(A-H)** GSE41328, GSE44076, GSE110223 and GSE110225 datasets were analyzed by MCODE for modular genes merging the top 10 genes of Degree values and removing duplicate values to obtain ferroptosis-related DEGs.

Supplementary Figure 5
**(A-E)** Statistical analysis of immunohistochemistry of key genes. **P* < 0.05, ***P* < 0.01 and ****P* < 0.001.

Supplementary Figure 6 The correlation of five core genes in the TIMER database in colon cancer.
