# Supplementary figures and images for: Effective prediction of potential ferroptosis critical genes in clinical colorectal cancer
Source: Front Oncol. 2022 Oct 17;12:1033044. doi: 10.3389/fonc.2022.1033044 (PMC9619366; doi:10.3389/fonc.2022.1033044)

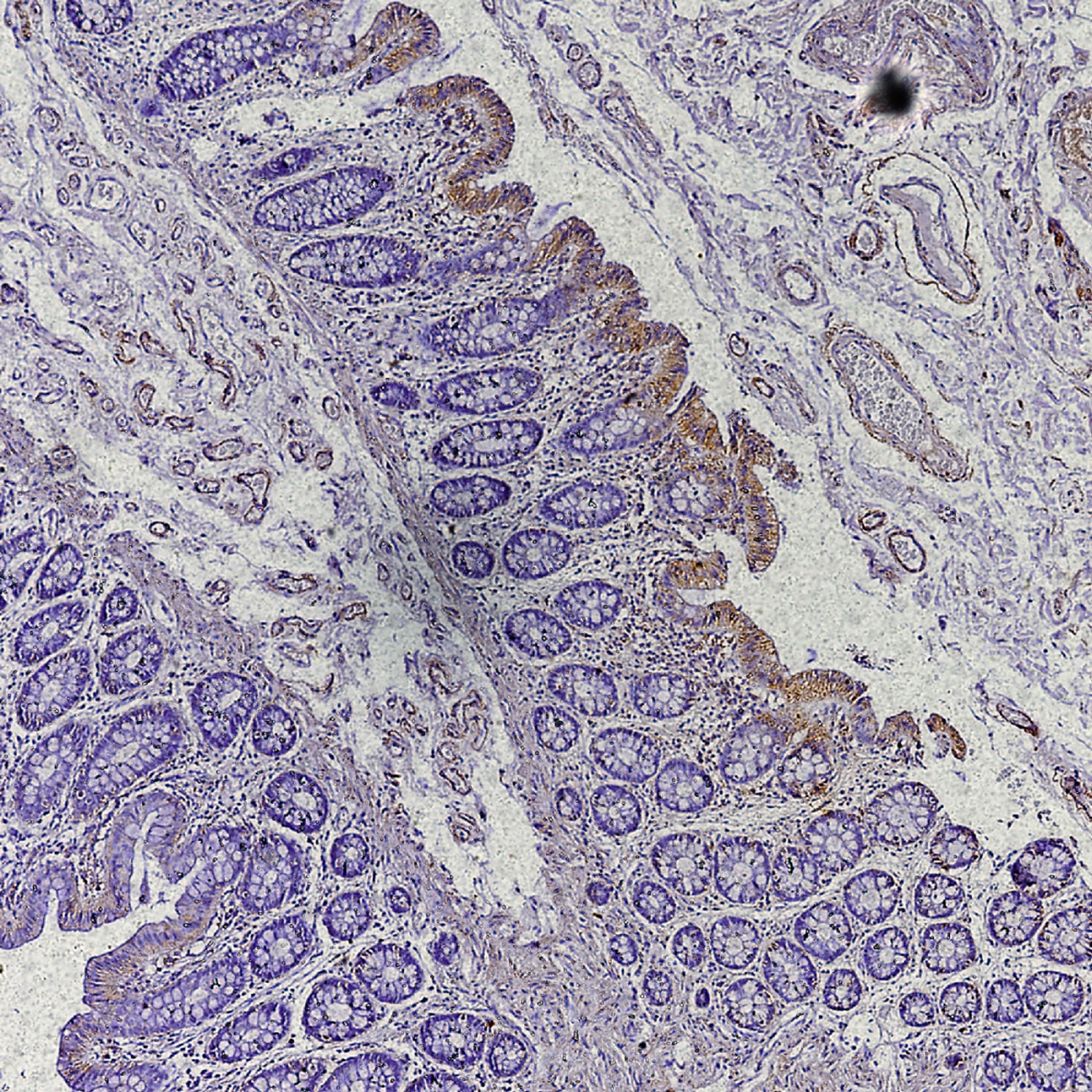

Supplement: Supplementary file 9 [file DataSheet_8.zip › IHC-ó±/ELEVL1.tif]

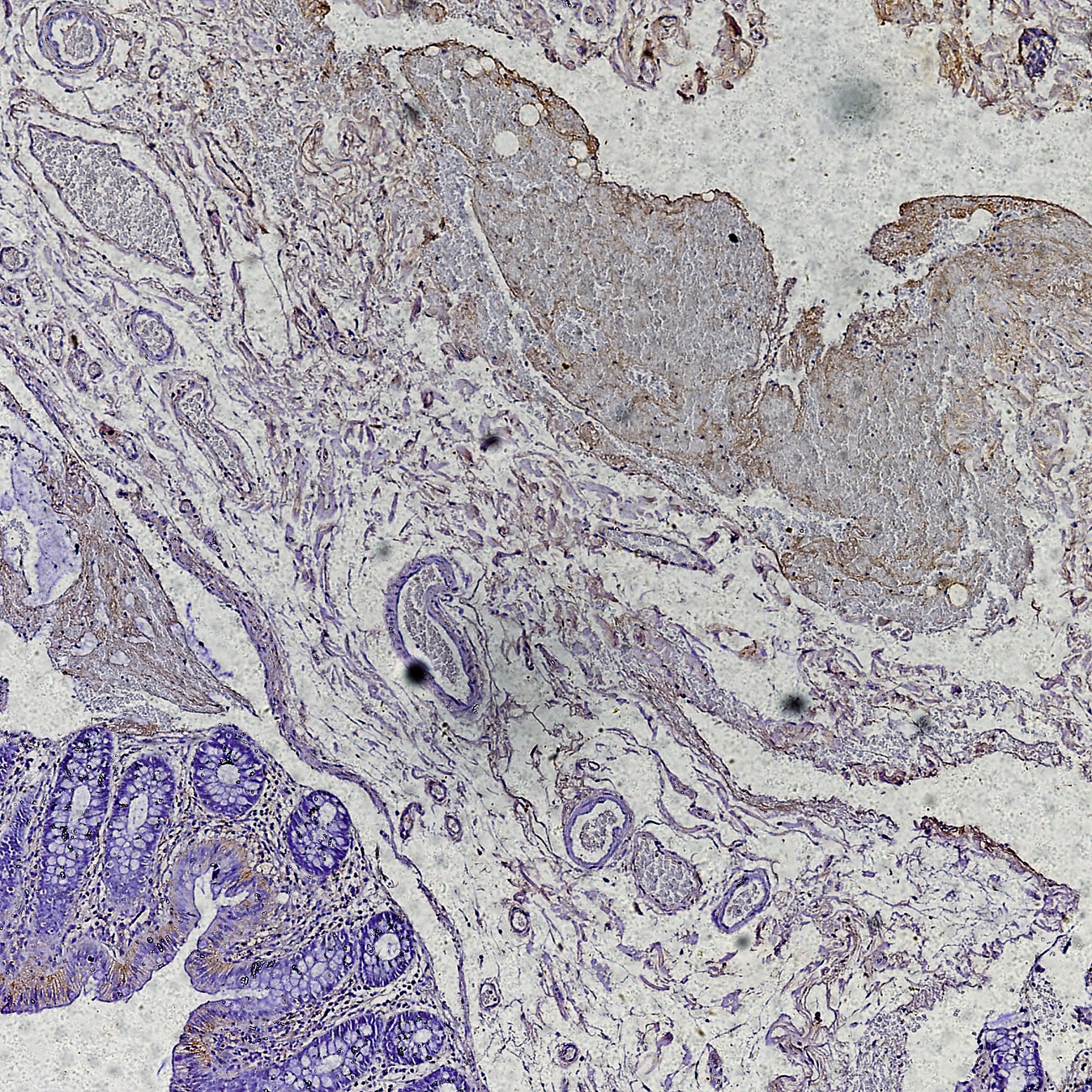

Supplement: Supplementary file 9 [file DataSheet_8.zip › IHC-ó±/EPAS1.tif]

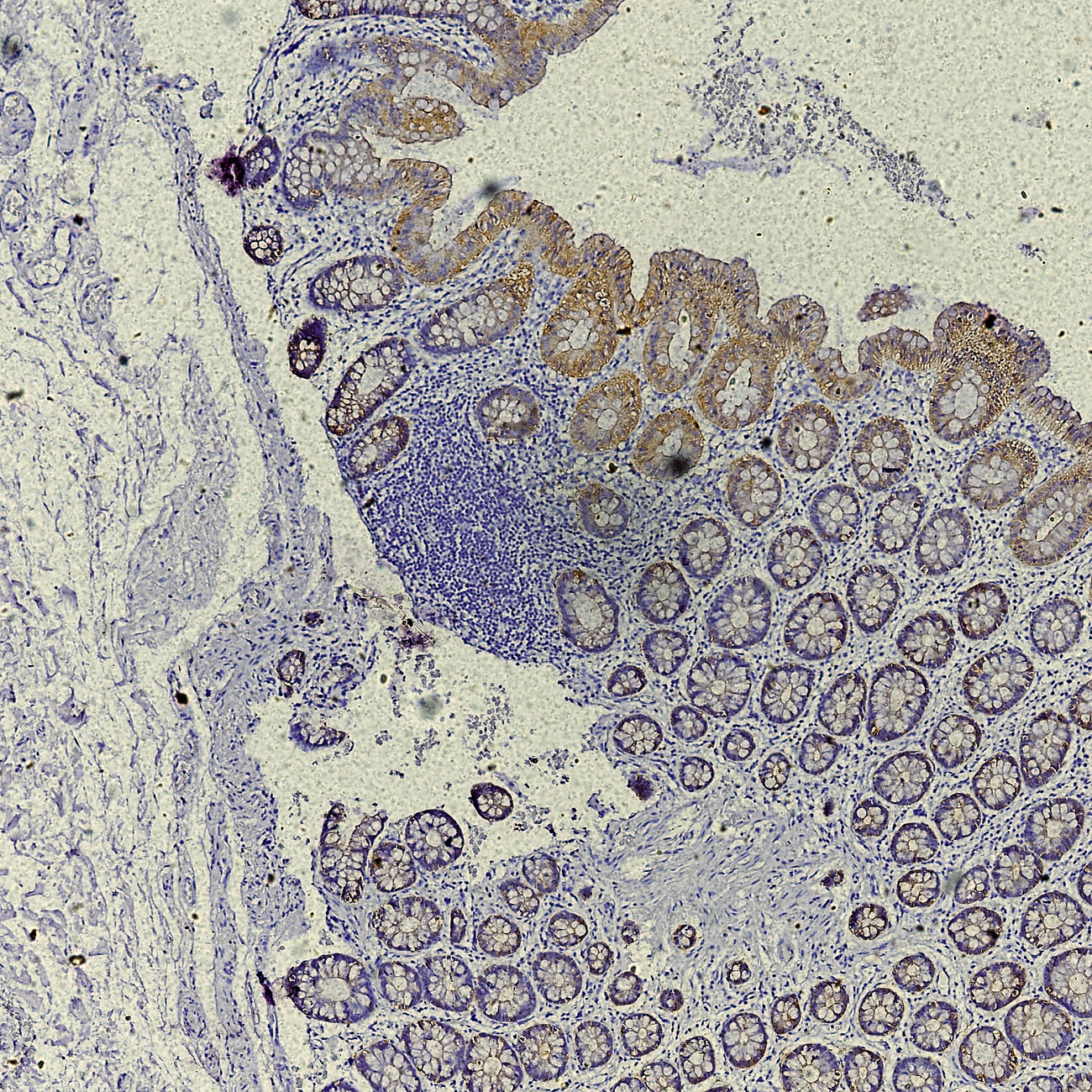

Supplement: Supplementary file 9 [file DataSheet_8.zip › IHC-ó±/GPX2.tif]

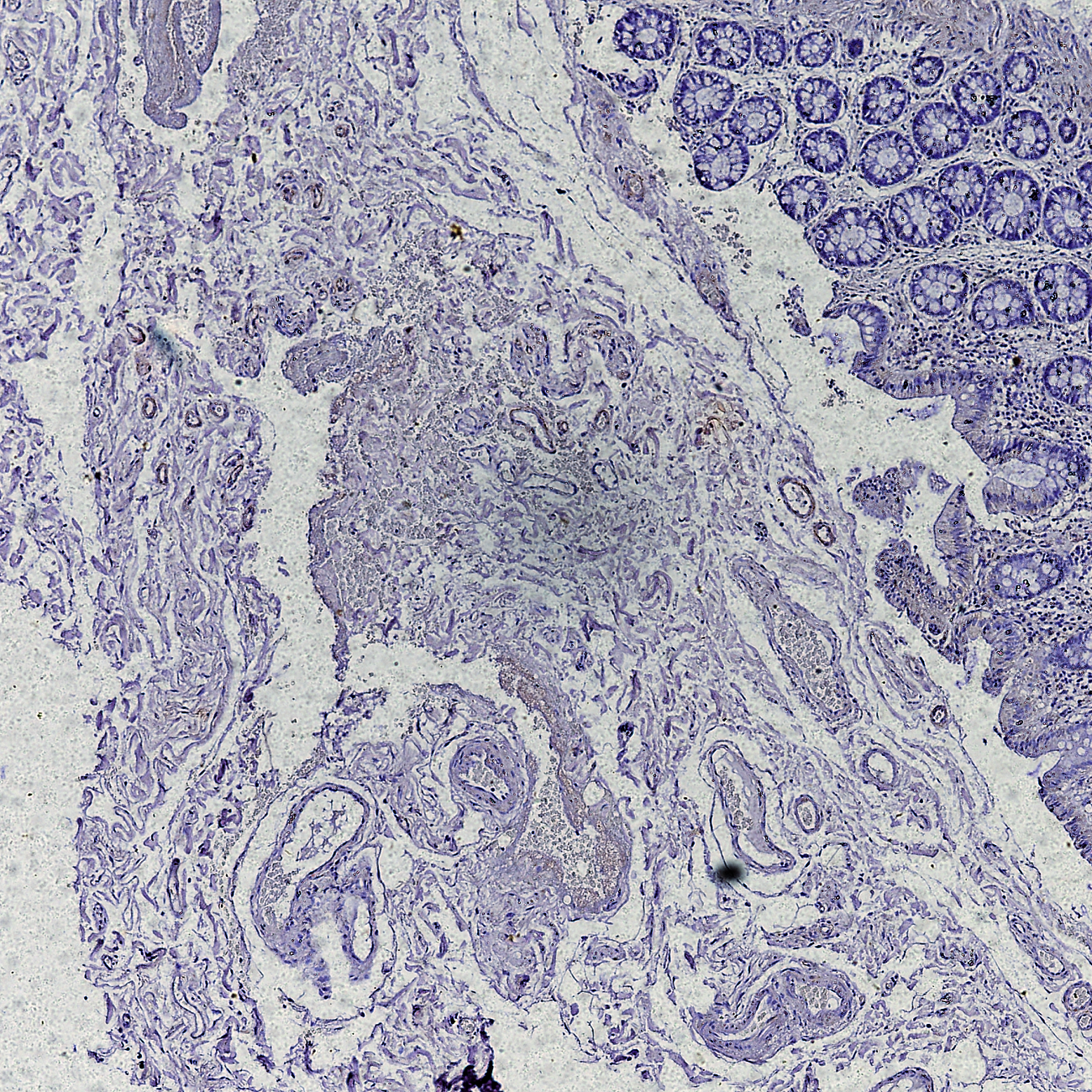

Supplement: Supplementary file 9 [file DataSheet_8.zip › IHC-ó±/HMGB1.tif]

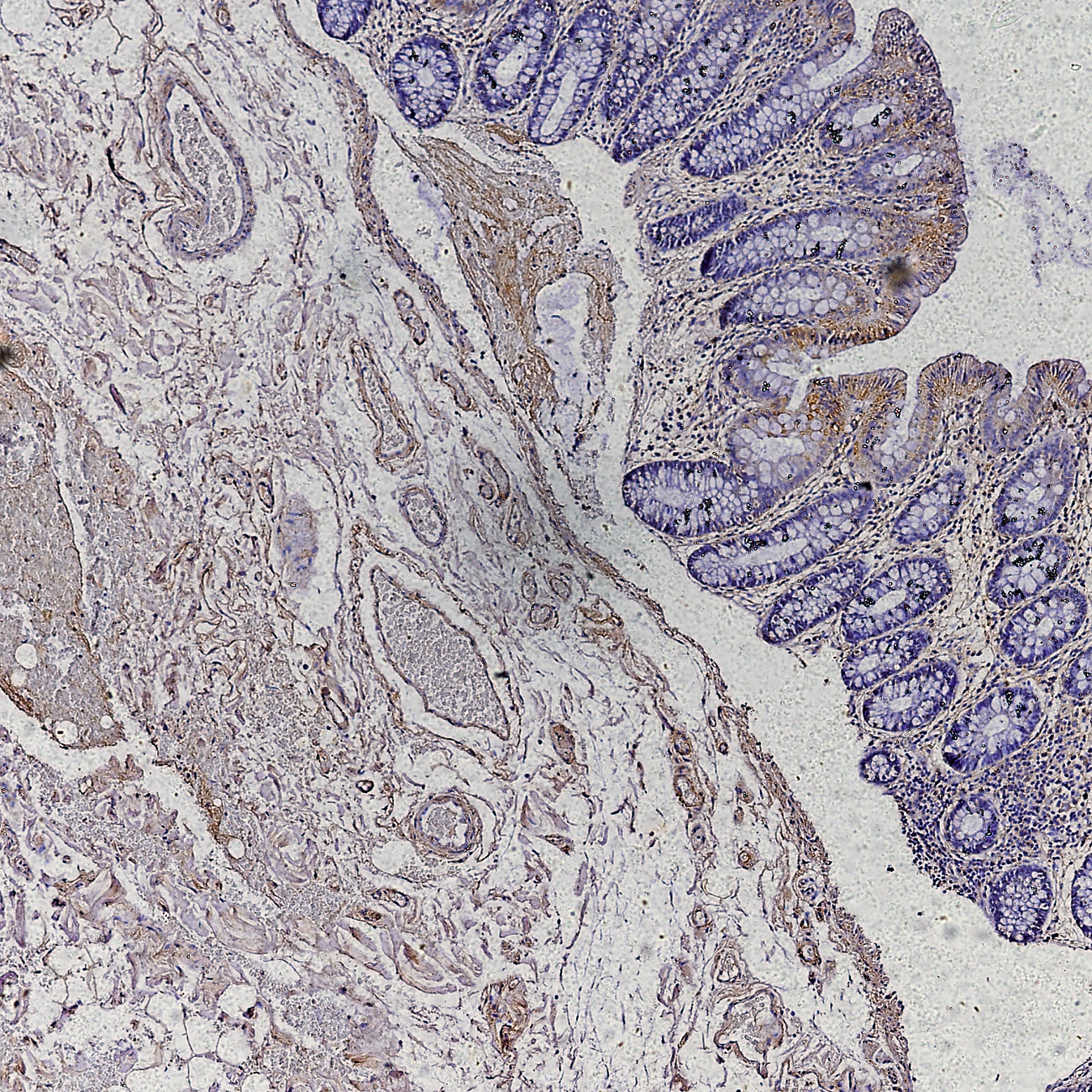

Supplement: Supplementary file 9 [file DataSheet_8.zip › IHC-ó±/SLC7A5.tif]

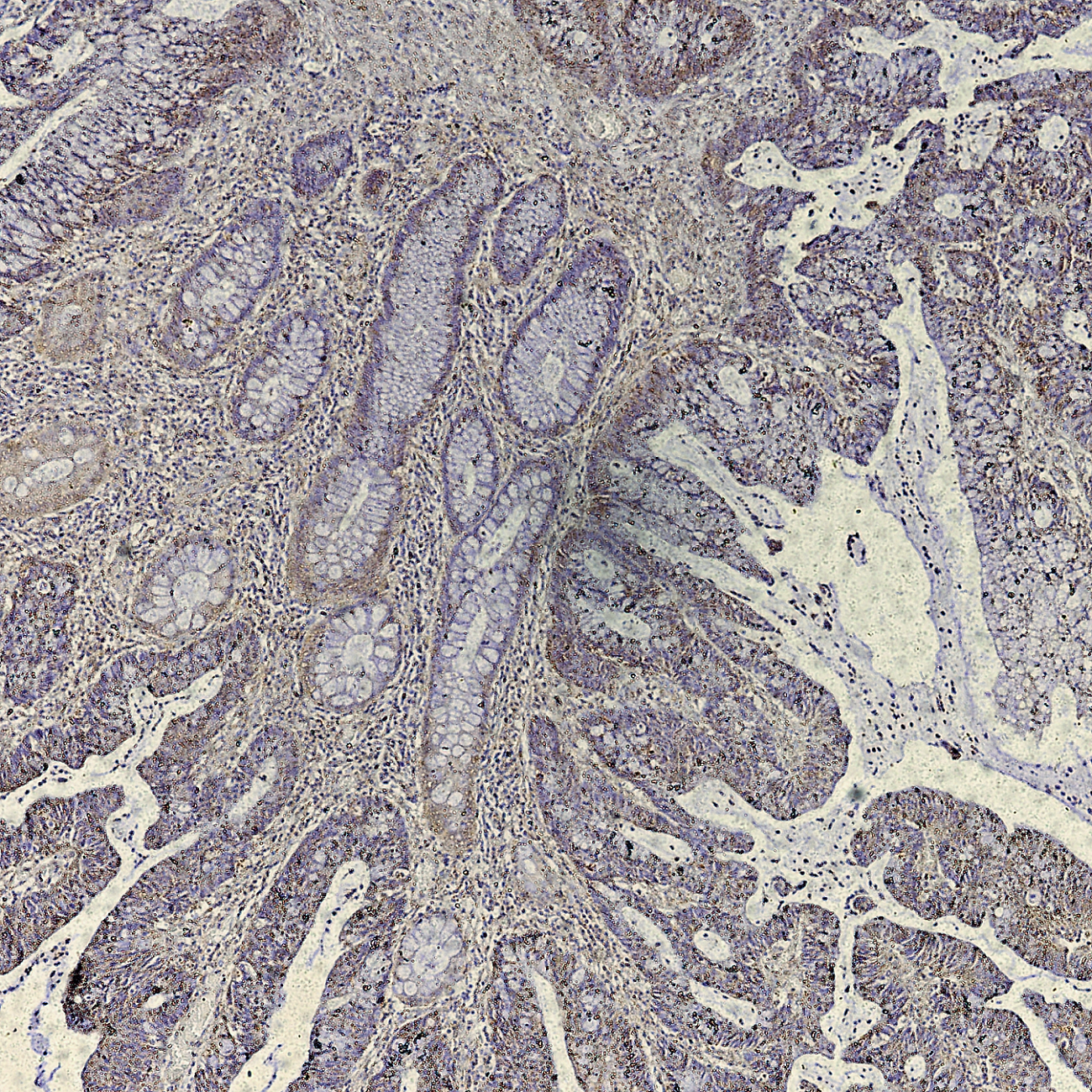

Supplement: Supplementary file 10 [file DataSheet_9.zip › IHC-ó≥/ELAVL1.tif]

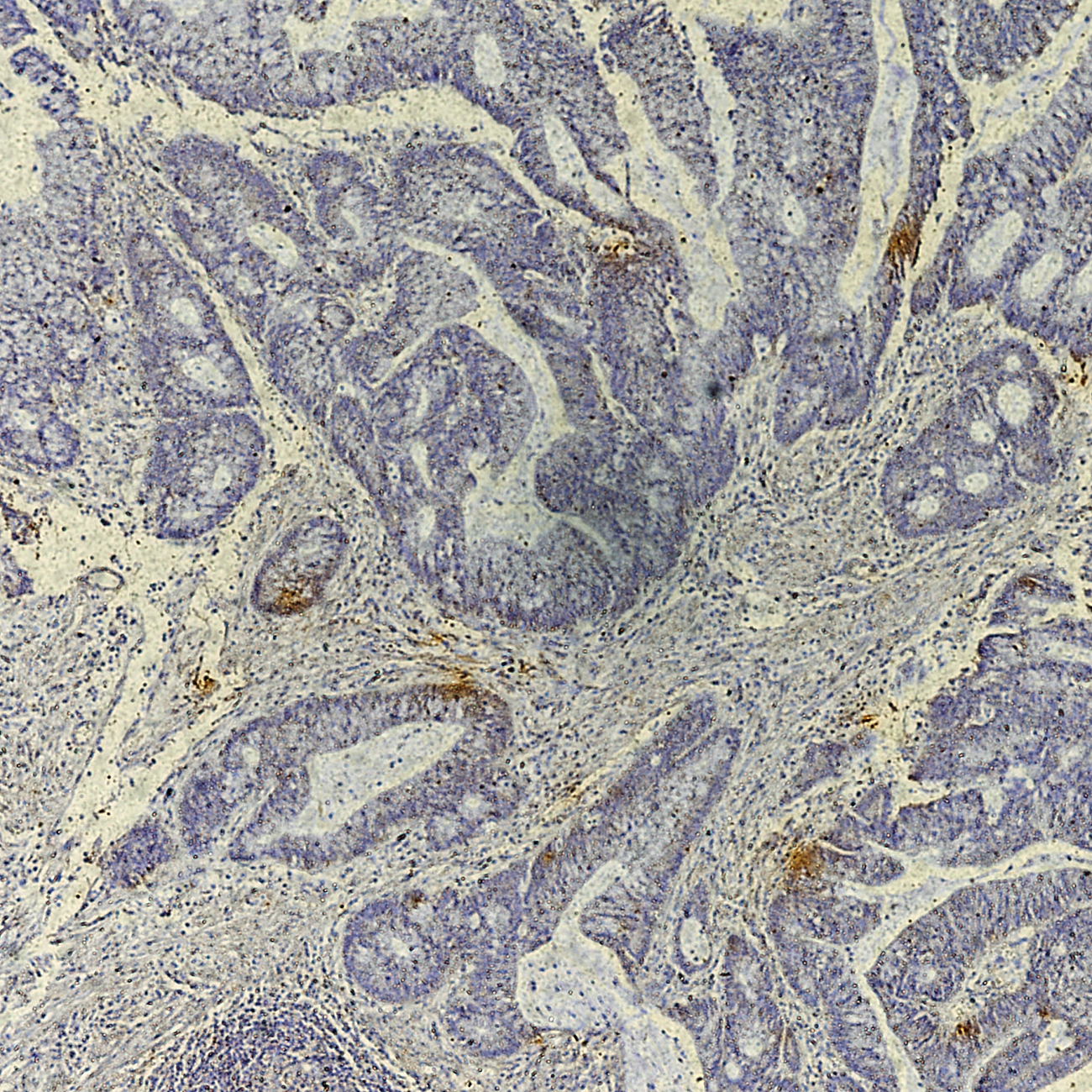

Supplement: Supplementary file 10 [file DataSheet_9.zip › IHC-ó≥/EPAS1.tif]

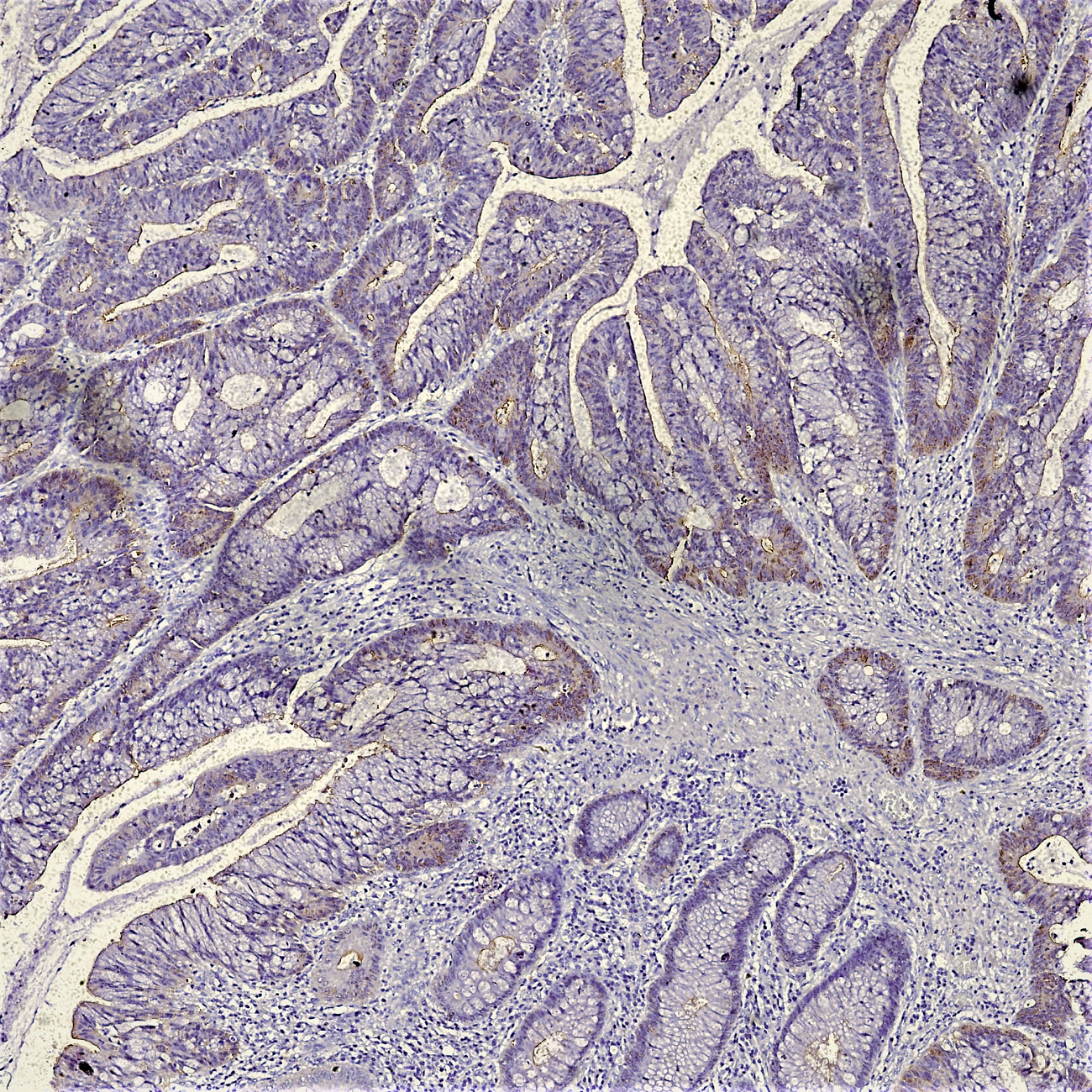

Supplement: Supplementary file 10 [file DataSheet_9.zip › IHC-ó≥/GPX2.tif]

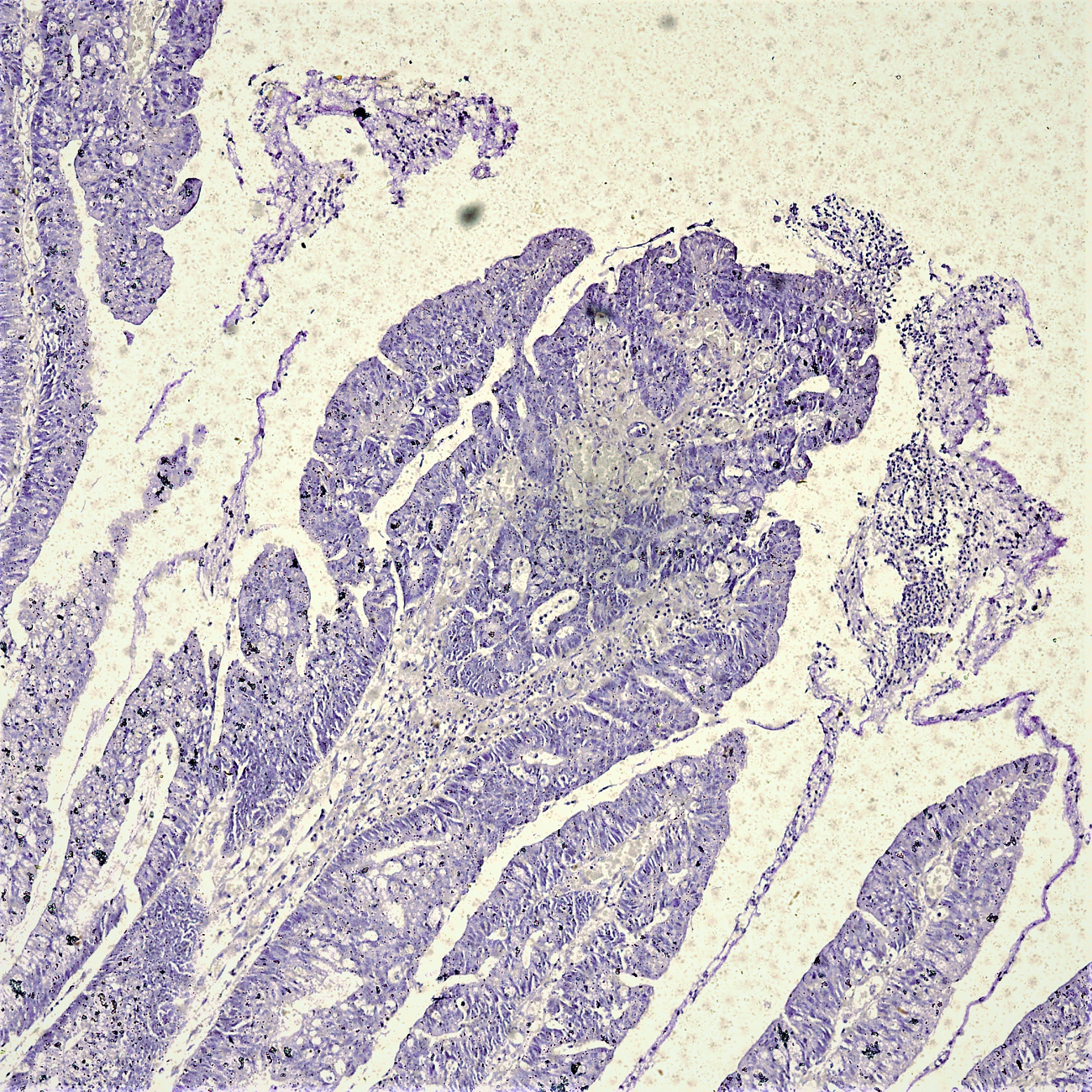

Supplement: Supplementary file 10 [file DataSheet_9.zip › IHC-ó≥/HMGB1.tif]

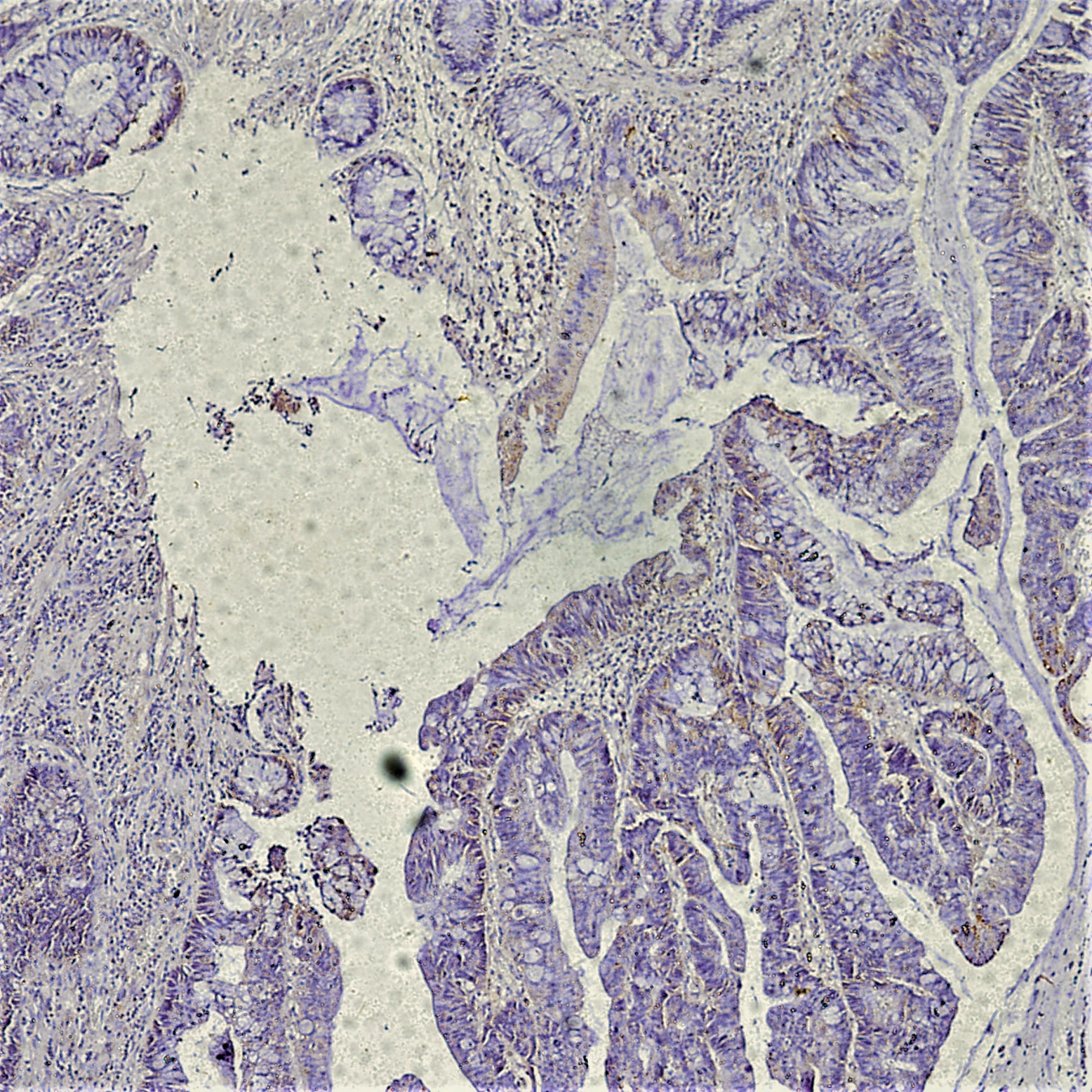

Supplement: Supplementary file 10 [file DataSheet_9.zip › IHC-ó≥/SLC7A5.tif]

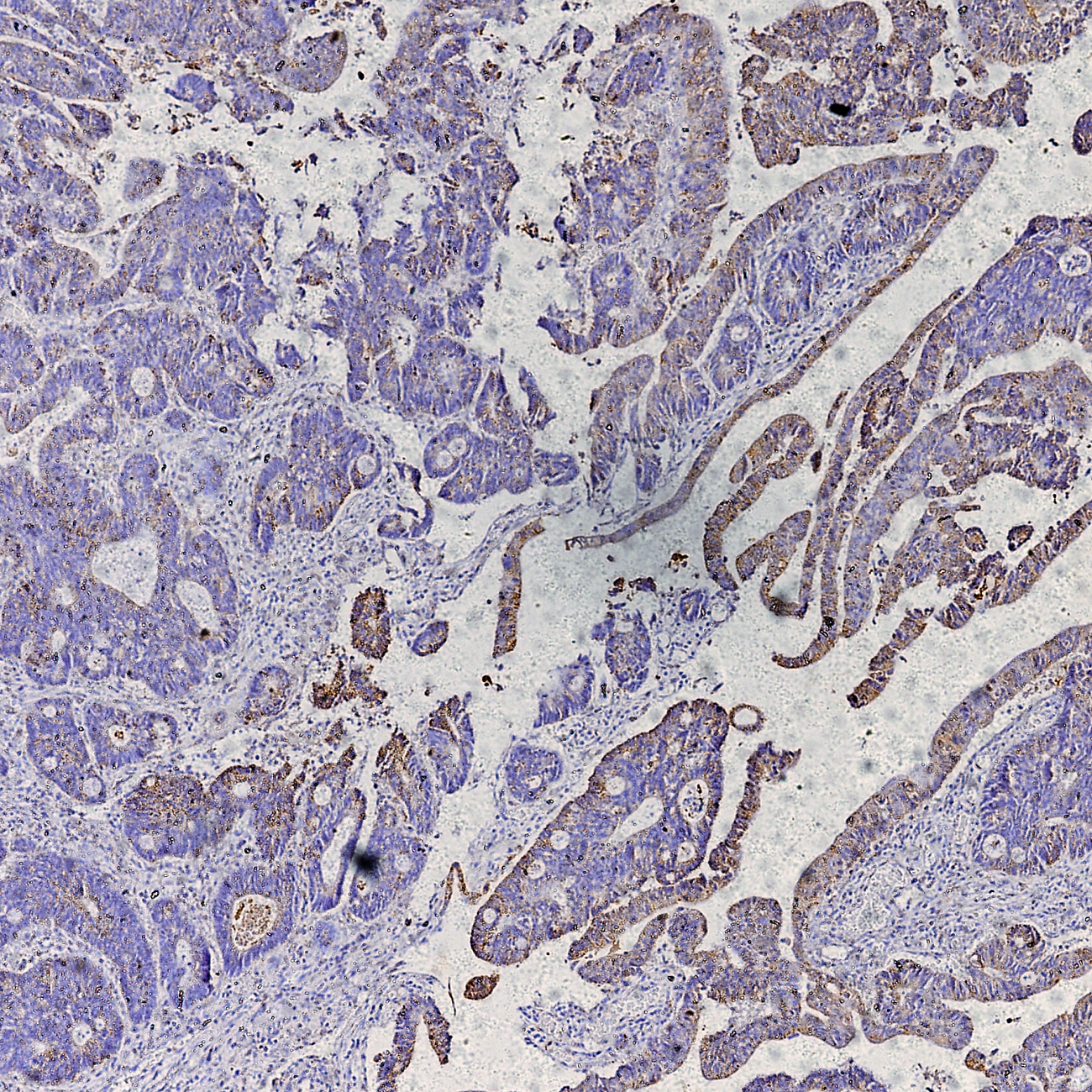

Supplement: Supplementary file 11 [file DataSheet_10.zip › IHC-ó≤/ELAVL1.tif]

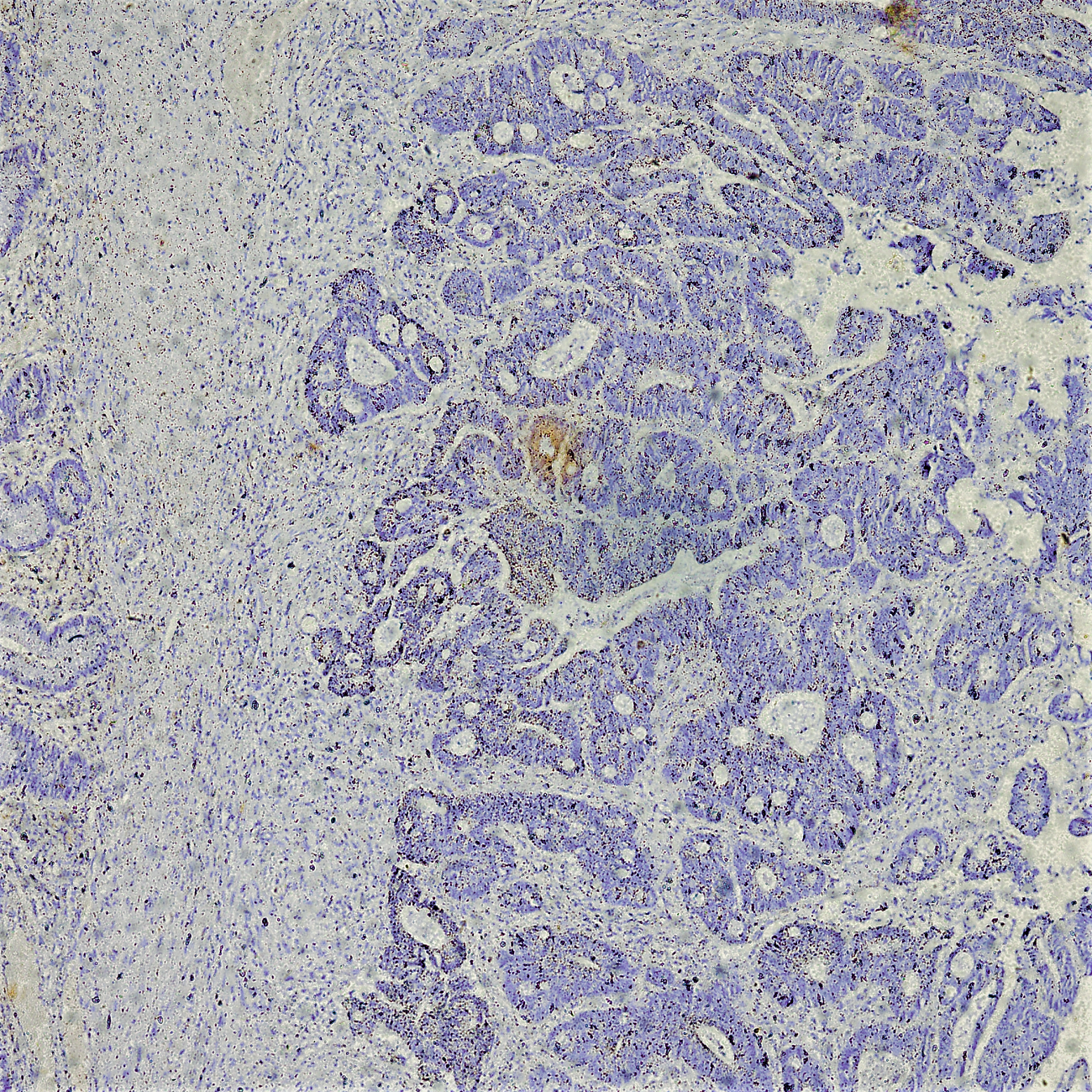

Supplement: Supplementary file 11 [file DataSheet_10.zip › IHC-ó≤/EPAS1.tif]

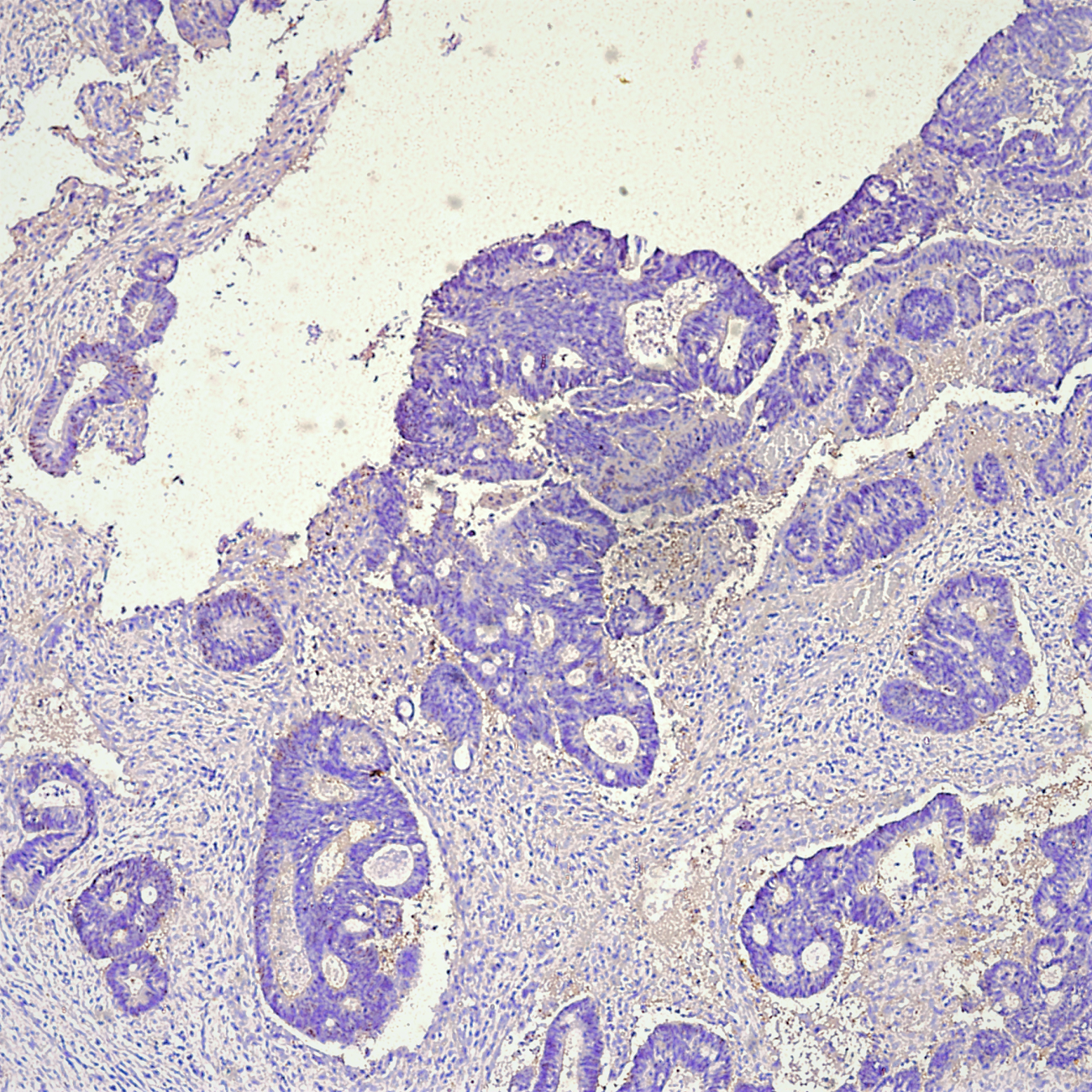

Supplement: Supplementary file 11 [file DataSheet_10.zip › IHC-ó≤/GPX2.tif]

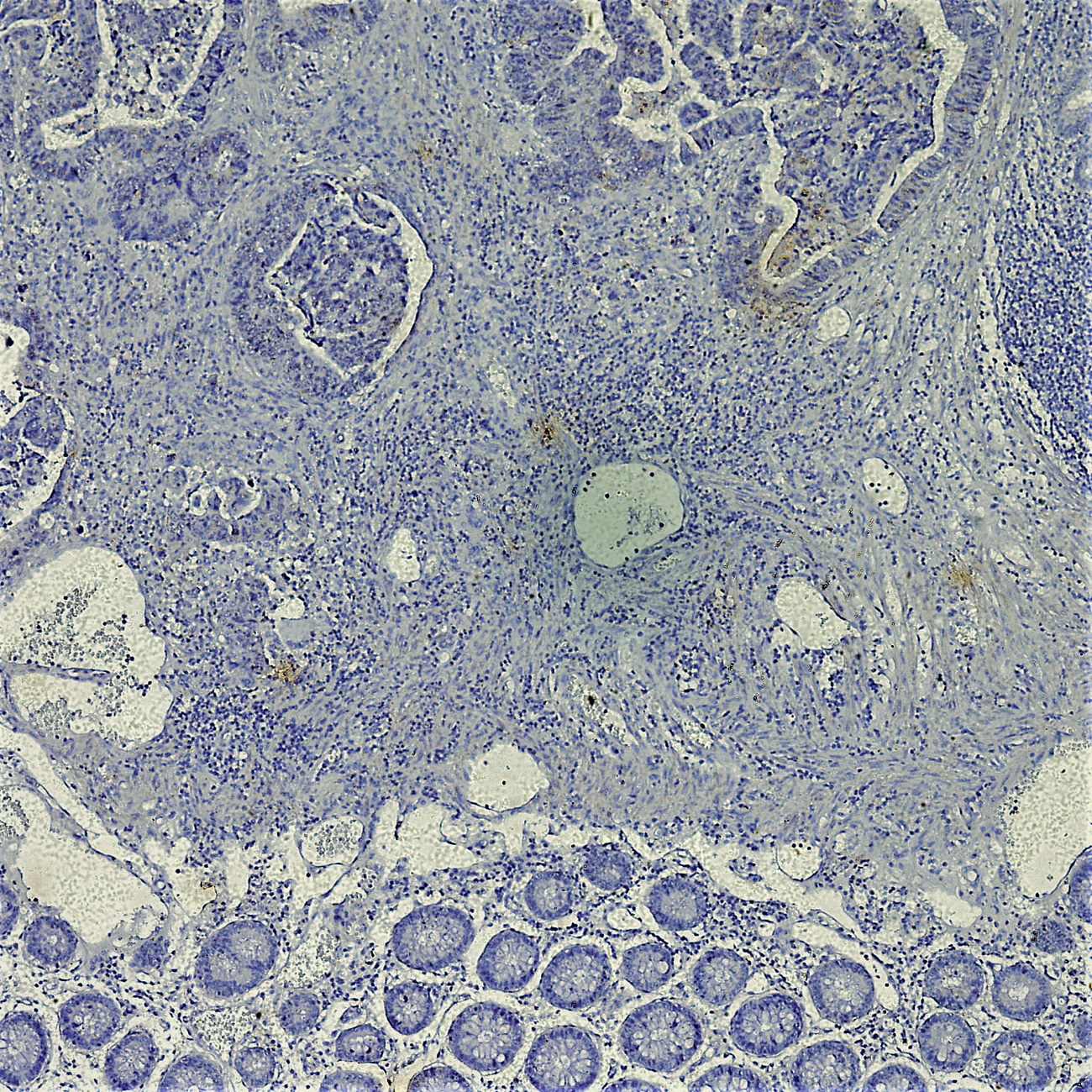

Supplement: Supplementary file 11 [file DataSheet_10.zip › IHC-ó≤/HMGB1.tif]

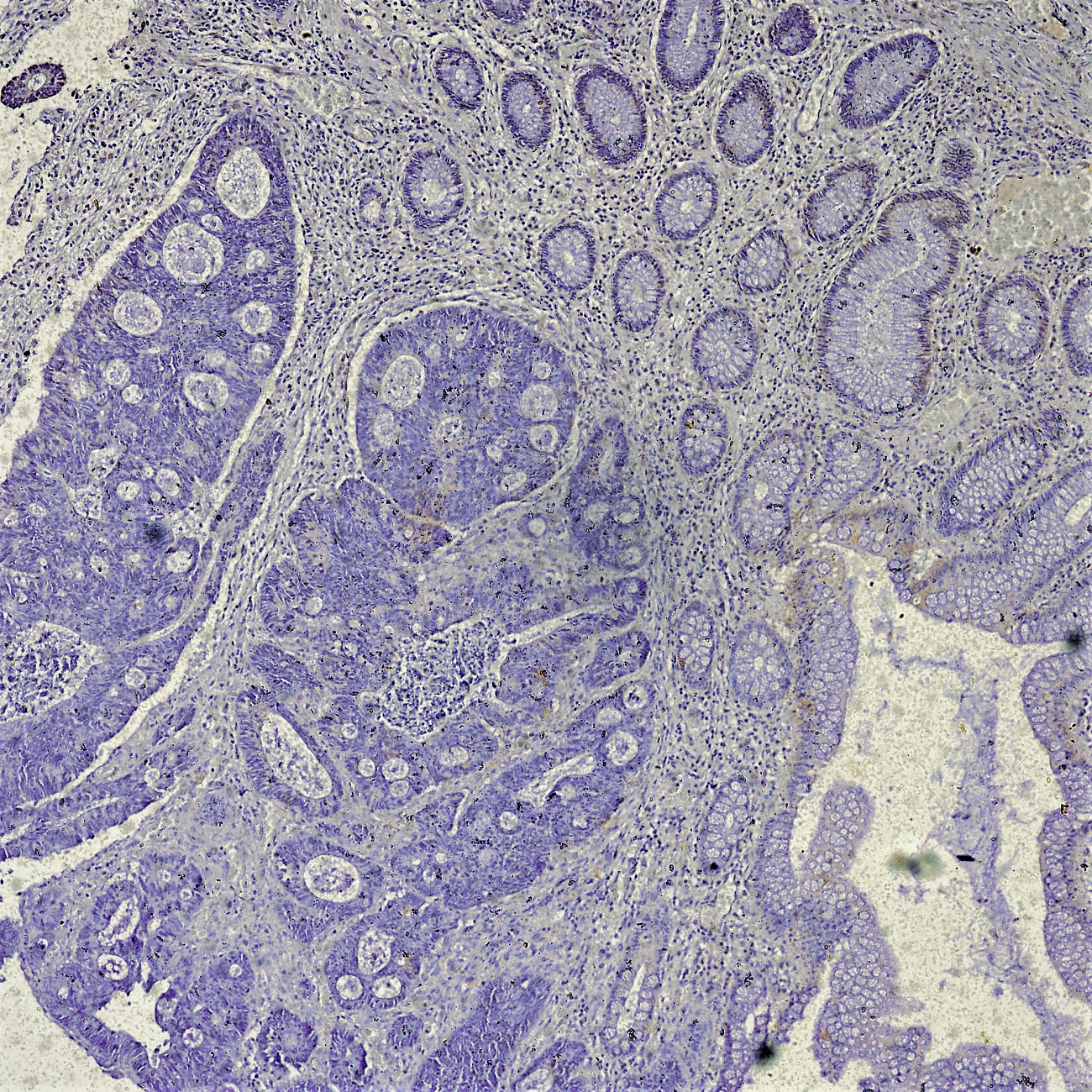

Supplement: Supplementary file 11 [file DataSheet_10.zip › IHC-ó≤/SLC7A5.tif]

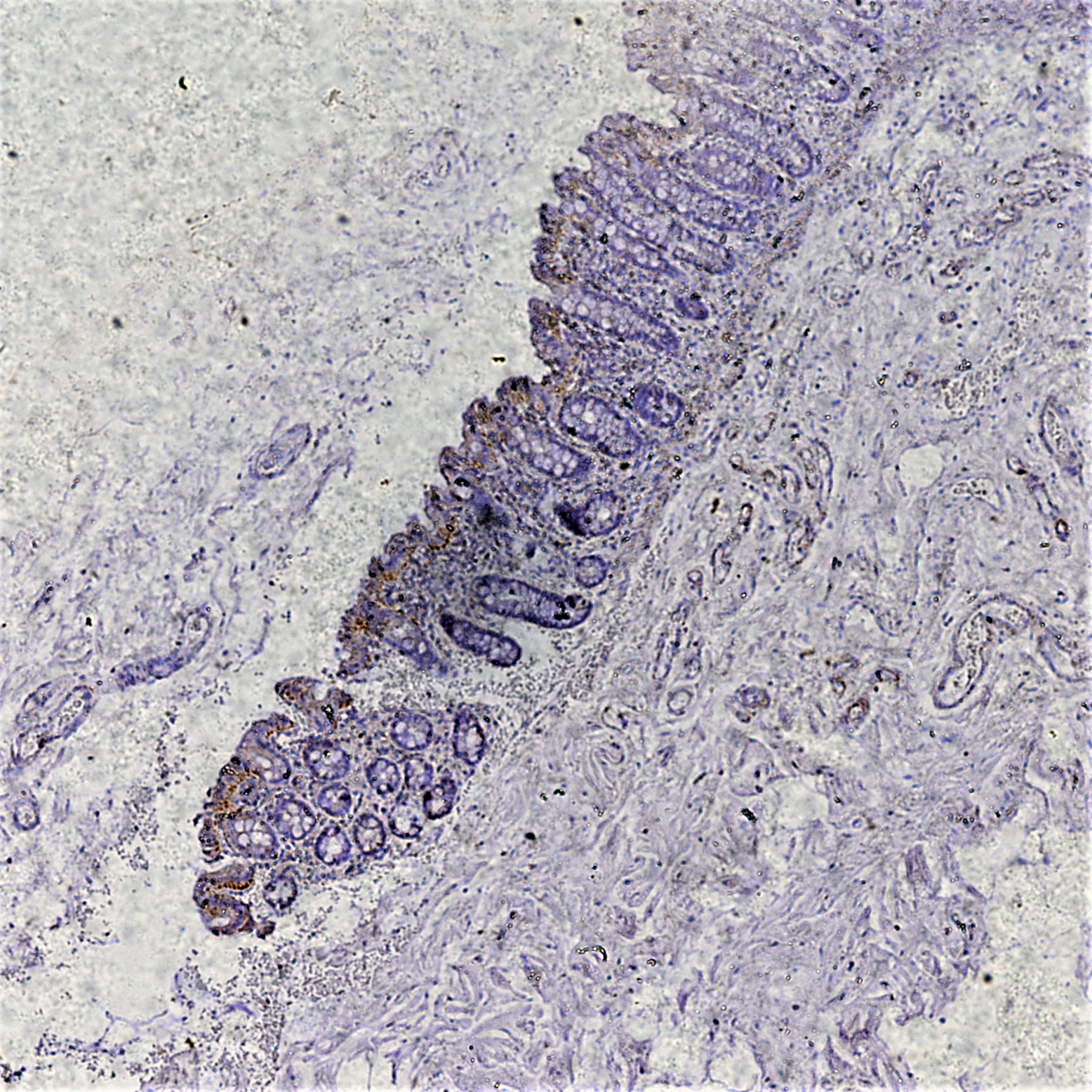

Supplement: Supplementary file 12 [file DataSheet_11.zip › IHC-normal/ELAVL1.tif]

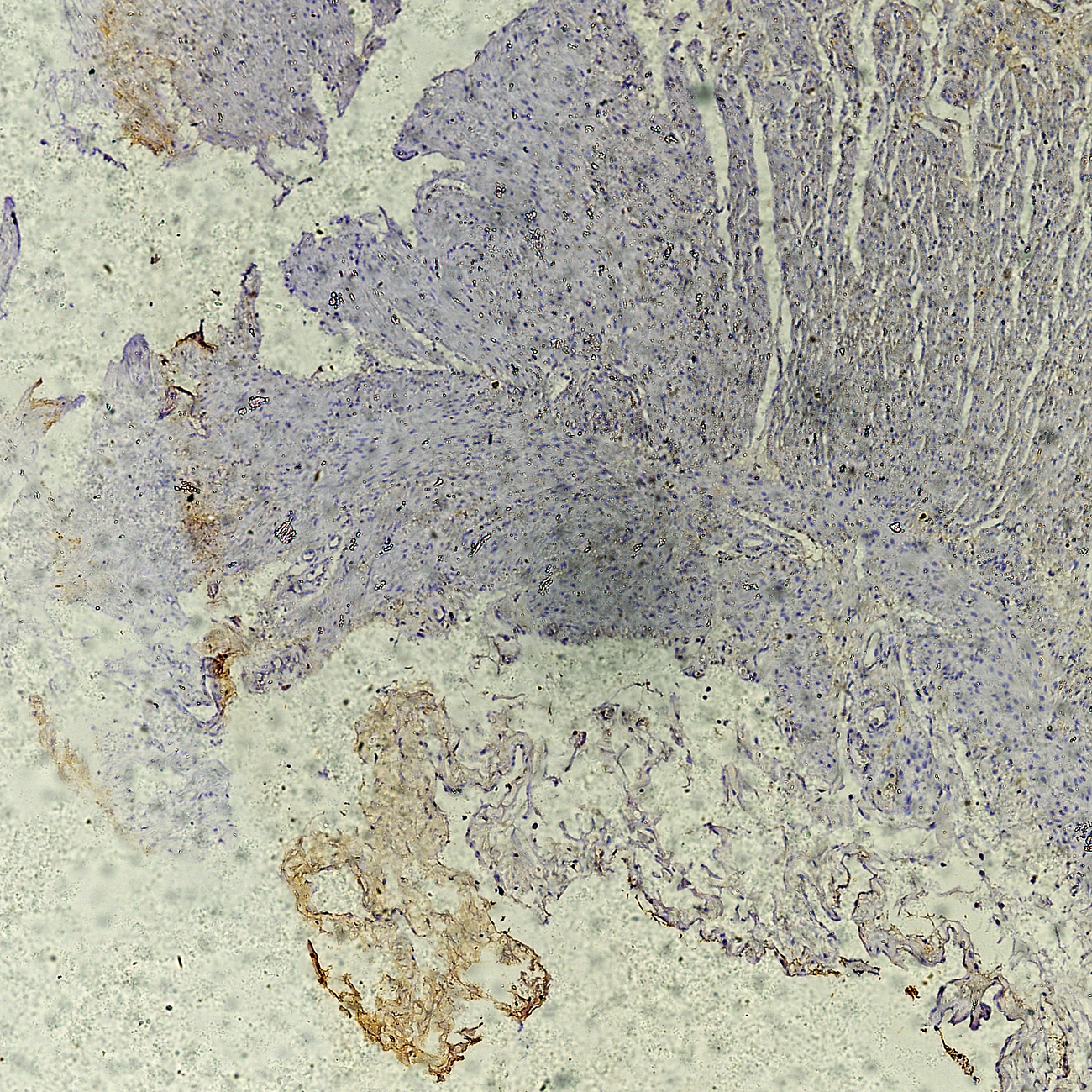

Supplement: Supplementary file 12 [file DataSheet_11.zip › IHC-normal/EPAS1.tif]

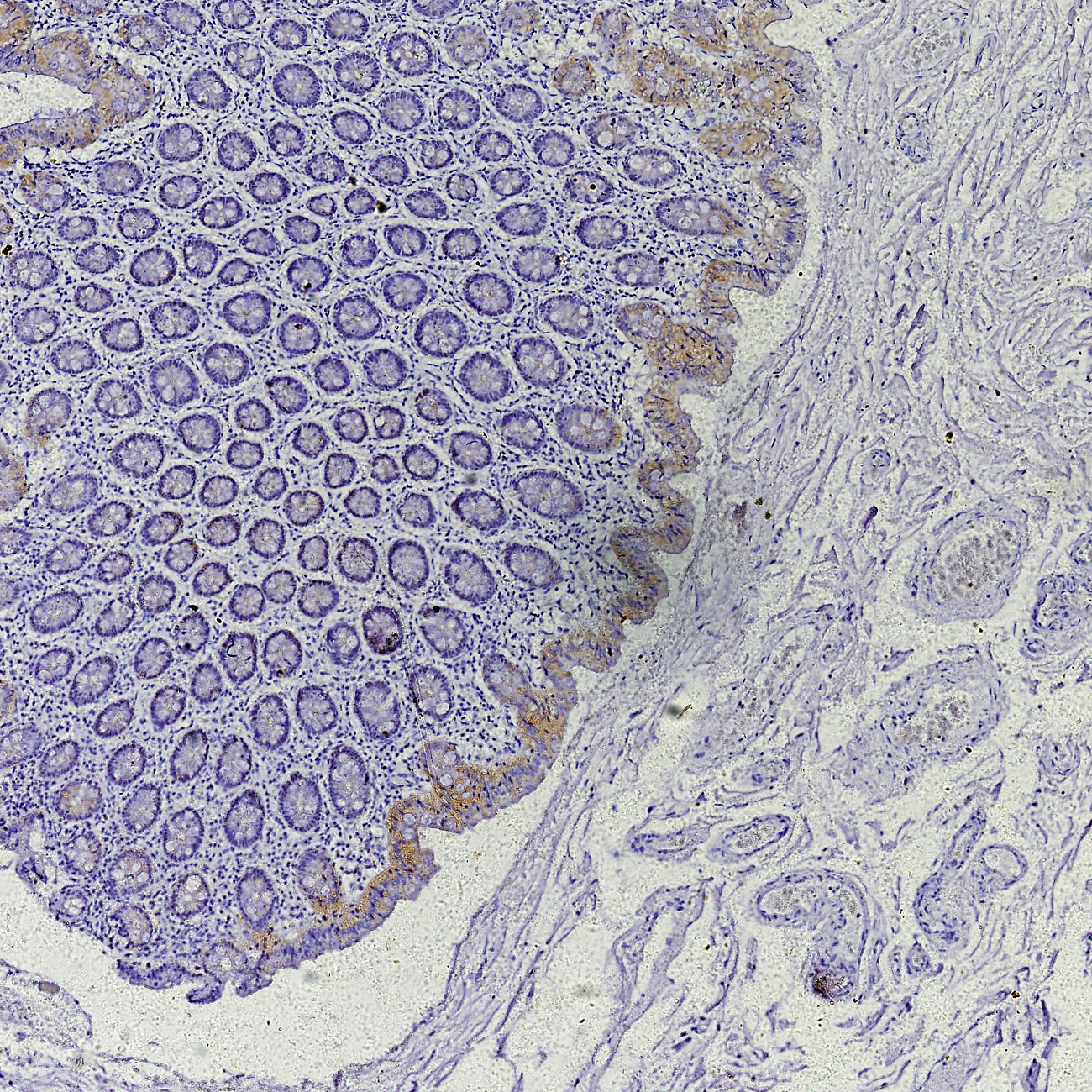

Supplement: Supplementary file 12 [file DataSheet_11.zip › IHC-normal/GPX2.tif]

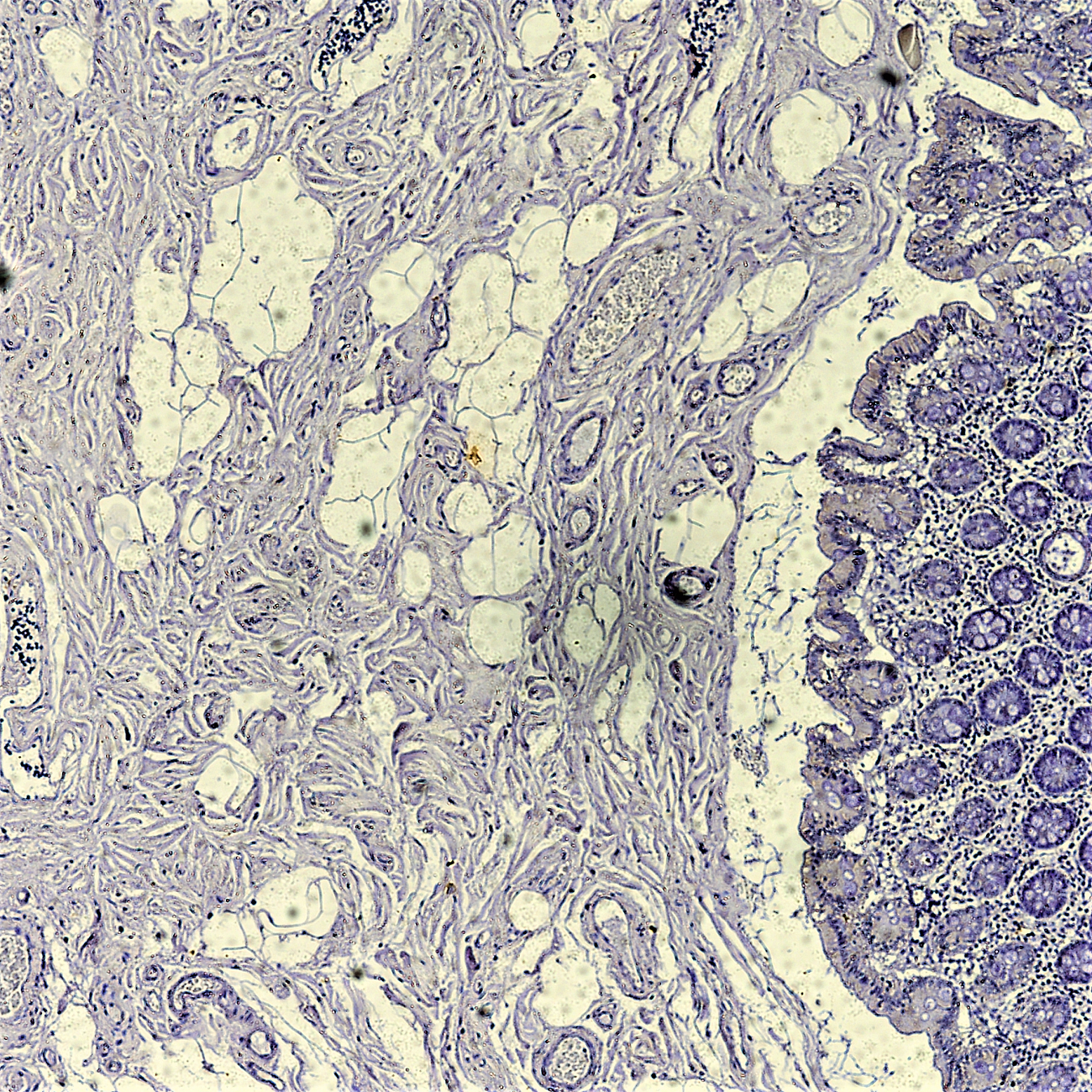

Supplement: Supplementary file 12 [file DataSheet_11.zip › IHC-normal/HMGB1.tif]

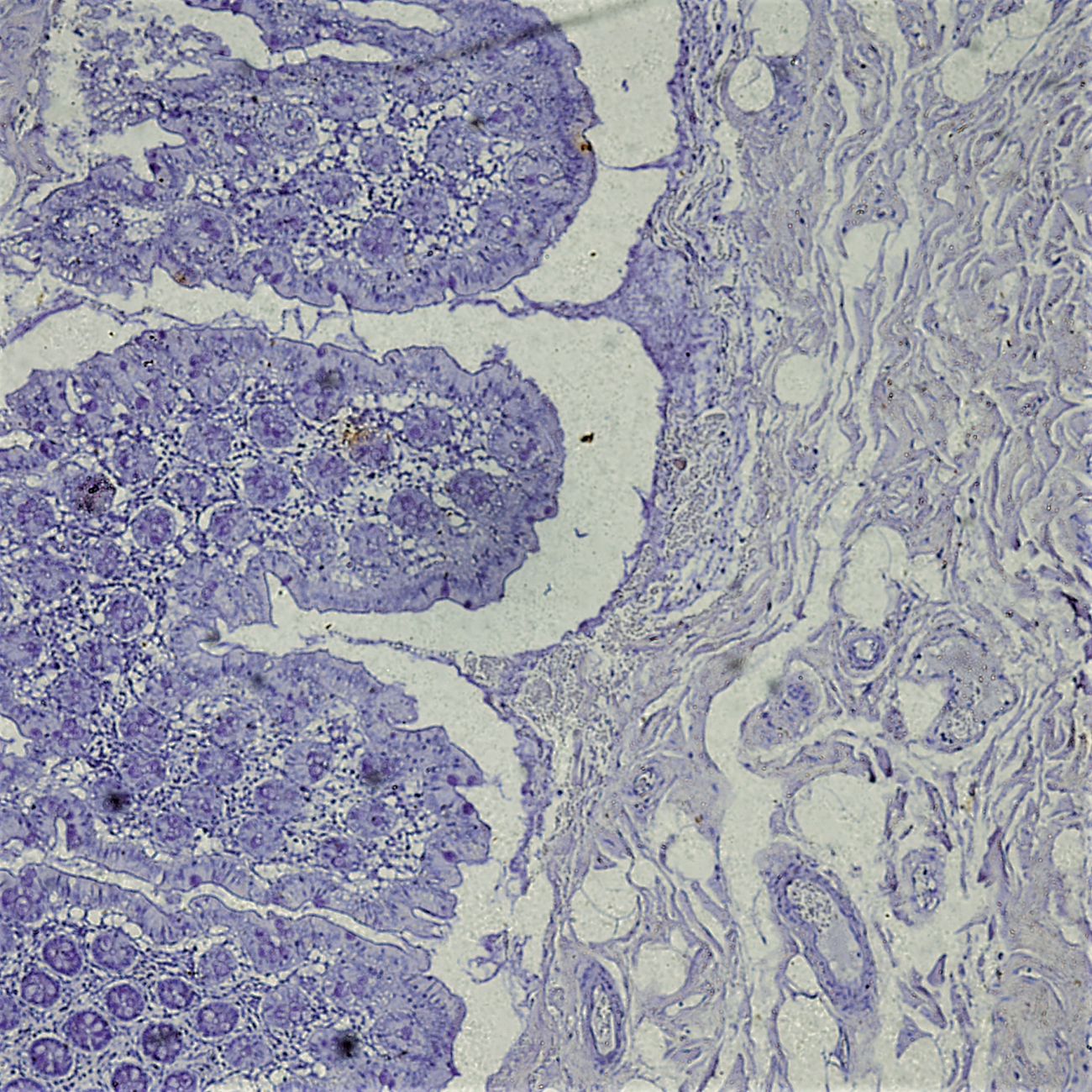

Supplement: Supplementary file 12 [file DataSheet_11.zip › IHC-normal/SLC7A5.tif]
